# Shaoyao Decoction reduced T lymphocyte activation by regulating of intestinal flora and 5-hydroxytryptamine metabolism in ulcerative colitis

**DOI:** 10.1186/s13020-024-00958-2

**Published:** 2024-06-15

**Authors:** Jianhua Zhen, Yini Li, Yunan Zhang, Yali Zhou, Lu Zhao, Guangrui Huang, Anlong Xu

**Affiliations:** 1 School of Life Sciences, Beijing University of Chinese Medicine, Beijing, 100029 China; 2 State Key Laboratory of Bio-Control, Department of Biochemistry, School of Life Sciences, Sun Yat-Sen University, Guangzhou, 510006 China

**Keywords:** Shaoyao Decoction, Ulcerative colitis, T lymphocyte, 5-hydroxytryptamine, Butyric acid, Intestinal flora

## Abstract

**Background:**

Shaoyao Decoction (SYD) is a widely recognized herbal formula utilized in traditional Chinese medicine for the treatment of diarrhea. Although it has demonstrated significant effectiveness in clinical practice for treating ulcerative colitis, the precise mechanisms by which it operates remain largely elusive.

**Methods:**

The active ingredients of SYD were obtained by ultra performance liquid chromatography tandem mass spectrometry (UPLC-MS/MS), which were used to explore the potential pharmacological mechanism based on TCMSP (Traditional Chinese Medicine Systems Pharmacology Database and Analysis Platform) and PANTHER (Protein Analysis Through Evolutionary Relationships) classification system. In a mouse model of dextran sulfate sodium (DSS)-induced colitis, mRNA sequencing, 16S rDNA sequencing and targeted metabolomics techniques were used to elucidate the mechanisms of SYD, and immunohistochemistry, immunofluorescence, enzyme linked immunosorbent assay, real time quantitative polymerase chain reaction and western blot were used to test the key targets. In addition, QGP-1 and H9 cells were performed to validate the discoveries from the animal experiments.

**Results:**

In the mouse model of DSS-induced colitis, SYD effectively alleviated symptoms such as bloody stool, tissue damage, inflammation, intestinal flora dysbiosis and abnormal gene expression. Analyses of both differential expressed genes in colonic tissue and predicted 16S rDNA genes, as well as the analyses of targeted genes from TCMSP based on the active ingredients in UPLC-MS/MS of SYD, uncovered the enrichment of pathways involved in the biosynthesis and degredation of 5-hydroxytryptamine (5-HT). Interestingly, SYD suppressed the relative abundance of key genes in 5-HT synthesis, Tph1(Tryptophan hydroxylase 1) and Ddc (Dopa decarboxylase), in faeces from DSS-induced mice, leading to a reduction in the concentration of fecal 5-HT. Moreover, SYD augmented the production of butyric acid. Subsequently, increasing butyric acid influenced the metabolism of 5-HT in the organism through G protein-coupled receptor 43 by impeding its synthesis, facilitating its transport and degredation. These findings were additionally corroborated in a model utilizing enterochromaffin cell (QGP-1 cells). Furthermore, reduced levels of 5-HT hindered the activation of T lymphocytes (H9 cells) via the PKC (Protein kinase C) and NF-κB (Nuclear factor kappa-B) signaling pathways, by means of HTR1A (5-HT receptor 1A) and HTR3 (5-HT receptor 3). Additionally, diminished secretion of 5-HT resulted in reduced secretion of associated cytokines, thereby alleviating inflammation in the colon.

**Conclusion:**

Through modulation of T lymphocyte activation mediated by 5-HT metabolism in the local colon via the intestinal flora and its metabolite, SYD effectively mitigated colonic inflammation in DSS-induced mice.

**Supplementary Information:**

The online version contains supplementary material available at 10.1186/s13020-024-00958-2.

## Background

Ulcerative colitis (UC) is a prevalent inflammatory bowel disease characterized by chronic non-specific inflammation in the rectal and colonic mucosa and submucosa. It presents with symptoms such as recurrent abdominal pain, diarrhea, mucus pus and bloody stool, with an unclear etiology. Prior studies have indicated that genetic, environmental and psychological factors contribute to mucosal barrier damage, neuroendocrine dysfunction and immune disorders, which collectively contribute to the progression of UC [1–3].

Shaoyao Decoction (SYD), a widely recognized and efficacious Traditional Chinese Medicine (TCM) formula, is commonly used for the treatment of diarrhea. It comprises *baicalensis*, *coptis*, *paeonia lactiflora*, *angelica*, *aucklandiae radix*, *areca*, *rhubarb*, *cinnamon* and *liquorice*. Some of these herbs have been scientifically shown to mitigate local intestinal inflammation in UC through the regulation of signaling pathways involving nuclear factor kappa-B (NF-κB), notch and Janus kinase 2/signal transducer and activator of transcription 3 (JAK2/STAT3), while also affecting the intestinal flora and its metabolites [4, 5]. For example, paeoniflorin and baicalin, active constituents identified in SYD through reverse phase high-performance liquid chromatography analysis [6], have been found to possess anti-inflammatory properties by primarily inhibiting the activation of the NF-κB signaling pathway and influencing the composition of the intestinal flora [7, 8]. Furthermore, berberine, an additional bioactive compound present in SYD, promotes the production of butyric acid through the intestinal flora, thereby enhancing the synthesis of tight junction proteins like zona occludens-1 [9, 10]. However, owing to the intricate composition of the TCM herbal formula, the comprehensive and precise pharmacological mechanism of SYD remains elusive. Therefore, we conducted this exploratory research employing mRNA sequencing, 16S rDNA sequencing and targeted metabolomics in mice with dextran sulfate sodium (DSS)-induced colitis, and further validated the details using QGP-1 and H9 cells.

## Marerials and methods

### Ingredients and potential targeted genes of SYD in ultra performance liquid chromatography tandem mass spectrometry (UPLC-MS/MS)

The herbal mixture underwent two rounds of decoction, with each round lasting for 1 h. Subsequently, the resultant decoction was concentrated and dried under vacuum to yield a powdered form. Firstly, a small amount of SYD powder was transferred to a vial, diluted with 100-fold methanol (Sigma-Aldrich, MO, USA) and extracted by ultrasonication (ThermoFisher, MA, USA) for 30 min, then the extraction was filtered and 1.5 mL filtrate was used to analyze the ingredients of SYD with an Vanquish UPLC system (ThermoFisher, MA, USA) and Q-Exactive Orbitrap mass spectrometer (ThermoFisher, MA, USA). Sheath gas and auxiliary gas were both using nitrogen. The parameters during the positive ion detection mode were: capillary temperature, 400 ℃; spray voltage, 3.5 kV; sheath gas flow rate, 40 arb; aux gas flow rate, 20 arb; while the parameters during the negative ion detection mode were: capillary temperature, 350 ℃; spray voltage, 3.0 kV; sheath gas flow rate, 35 arb; aux gas flow rate, 10 arb. The full scan mode covered 200-2000 m/z, and the resolution was 70000 (full scan) and 17500 (MS/MS). Collision mode was high energy collision dissociation, with NCE30 and stepped NCE50% as the collision energy. The data were analyzed using Xcalibur software (V4.2, ThermoFisher, MA, USA). Furthermore, we quantified the key ingredients in SYD as the following procedure: the TCM formula powder was diluted with 80% methanol and vortexed by BeyoVortex^™^ (Beyotime, Beijing, CHN) for 5 min, then extracted by ultrasonication for 20 min. The solution after ultrasonic extraction was centrifuged at 13000 rpm at 4 ℃ for 10 min (ThermoFisher, MA, USA), and the supernatant was aspirated and diluted 100-fold with 50% methanol into a clean vial for UPLC-MS/MS, and the standard curve was prepared with 50% methanol aqueous solution at the same time. Subsequently, the prepared sample was analyzed with an Agilent ultrahigh performance liquid chromatography ABSCIEX Exion LC AD system (SCIEX, MA, USA) with a Waters XSelect HSS T3 Column (2.5 µm, 2.1 × 100 mm, Waters, MA, USA) at 40 ℃. Five µL sample was injected, and the flow rate was set at 0.3 mL/min. The mobile phase consisted of acetonitrile (A) and 0.05% formic acid (Sigma-Aldrich, MO, USA) in water (B). The gradient program was as follows: 0 min, A/B (15:85, v/v); 2 min, A/B (30:70, v/v); 6 min, A/B (60:40, v/v); 8 min, A; 10.1 min, A/B (15:85, v/v). To obtain the mass spectrometry (MS) and MS/MS data, we used an ABSCIEX QTRAP 5500 system (SCIEX, MA, USA). The following parameters were applied: spray gas, 55 psi; auxiliary heating gas, 55 psi; capillary temperature, 500 ℃; ion voltage, 4500 V-5500 V; curtain gas, 30 psi; collision gas, 9 psi. Furthermore, we screened all potential targeted genes associated with the ingredients of SYD in the UPLC-MS/MS analysis with the aid of TCMSP (Traditional Chinese Medicine Systems Pharmacology Database and Analysis Platform, https://old.tcmsp-e.com/tcmsp.php) [11]. Next, all the targeted genes were inputted into the PANTHER (Protein Analysis Through Evolutionary Relationships, http://www.pantherdb.org/) [12] classification system for the purpose of conducting pathway enrichment analysis. The results were presented in the form of a bubble chart in the Supplementary Figures.

### Animals and sampling

Colitis models were induced in male BALb/c mice weighing 17-19 g by administering 5% dextran sulfate sodium (DSS, Wako, Tokyo, Japan). Following the detection of occult blood in over 50% of the mice’s stool, they were then administered with various doses of SYD (low dose of Shaoyao Decoction, abbreviated as SYD-L: 8 g/kg/day; medium dose of Shaoyao Decoction, abbreviated as SYD-M: 16 g/kg/day; high dose of Shaoyao Decoction, abbreviated as SYD-H: 32 g/kg/day) or mesalazine (Mes, 0.5 g/kg/day, Etiasa, Shanghai, CHN). Following 7 days of treatment, serum, digesta in the colon (as fecal samples) and colonic tissue were collected for subsequent experiments.

### mRNA sequencing and the data processing

Colonic tissues were used to isolate total RNA through the utilization of Trizol reagent (Invitrogen Life Technologies, CA, USA). Subsequently, Poly(A) + RNA was purified using the NEBNext Poly(A) mRNA Magnetic Isolation Module (New England Biolabs, MA, USA). Qualified RNA samples underwent fragmentation and reverse transcription into cDNA employing the NEBNext Ultra RNA Library Prep kit for Illumina (New England Biolabs, MA, USA). RNA sequencing was performed on the Illumina Novaseq Platform (Illumina, CA, USA). The resulting clean data were aligned to the GRCm38 reference database (https://www.ensembl.org/) using HISAT2 (V2.1.0, Johns Hopkins University, MD, USA), followed by transcript assembly using StringTie (V1.3.3b, Johns Hopkins University, MD, USA). RNA expression levels were quantified using fragments per kilobase of transcript per million fragments mapped (FPKM). Principal coordinate analysis (PCoA) with Adonis analysis was used to exhibit the mRNA profiles. The differentially expressed genes (DEGs) were screened according to |Log_2_Fold change (FC)|≥ 1 and *P* ≤ 0.05, and displayed using volcano plot and heatmap. DEGs were further subjected to functional annotation and pathway analyses using the ENSEMBL knowledgebase (http://www.ensembl.org/index.html) and the Kyoto Encyclopedia of Genes and Genomes (KEGG, http://www.kegg.jp/) /PANTHER classification system [13], which were presented in bubble diagrams.

### 16S rDNA full-length sequencing and the data processing

Genomic DNA was extracted from the fecal samples using a Tiangen DNA kit (Tiangen, Beijing, CHN). Bacterial 16S rDNA was amplified using unique molecular identifier (UMI) primers and KAPA HiFi HotStart ReadyMix (Roche, Shanghai, CHN) with the universal primers 27F (5’-AGAGTTTGATCMTGGCTCAG-3’) and 1492R (5’-GGYTACCTTGTTACGACTT-3’). Following amplification, the amplicons were purified with Beckman Agencourt AMPure XP (Beckmancoulter, TX, USA) and quantified using a ThermoFisher Qubit dsDNA assay kit (ThermoFisher, MA, USA). The sequencing library was constructed by pooling equal amounts of purified amplicons and subjected to full 16S rDNA sequencing on the Illumina platform (Illumina, CA, USA). The paired reads derived from the original DNA fragments were merged using Illumina bcl2fastq conversion (V2.20, Illumina, CA, USA). The sequences were then analyzed using the Quantitative Insights Into Microbial Ecology (QIIME) software (V2.10, http://www.qiime.org). Operational taxonomic units (OTUs) were assigned at 99% similarity, and a representative sequence from the SILVA 132/16S rDNA database (V132, http://www.arb-silva.de) for bacteria was assigned to each OTU. Taxonomic data were generated using a Bayesian approach with a 99% cutoff value. Kruskal-Wallis analysis was performed to compare the diversity measures statistically. PCoA with Adonis analysis based on the Bray-Curtis distance was used to demonstrate the beta diversity of the intestinal flora. KEGG pathways were obtained using Phylogenetic Investigation of Communities by Reconstruction of Unobserved States (PICRUSt, V1.1.4, https://picrust.github.io/picrust/), and the functional difference among groups was displayed using linear discriminant analysis effect size (LEfSe) analysis with *P* ≤ 0.05 and LDA ≥ 2 as threshold. Spearman's correlation coefficient was used to assess the relationship between genera and the relative abundance of genes in the faeces.

### Targeted metabolomics for short-chain fatty acids (SCFAs)

Prepared stock solutions of acetic acid (Aladdin, Shanghai, CHN), propionic acid (Sigma-Aldrich, MO, USA), butyric acid (Sigma-Aldrich, MO, USA), isobutyric acid (Trc, YTO, CA), pentanoic acid (NU-CHEK, MN, USA), hexanoic acid (Dr.Ehrenstorfer, Augsburg, GER) and isovaleric acid (Yuanye, Shanghai, CHN) with a concentration of 1 mg/mL in distilled water (DW). These stock solutions were then diluted with water to the desired concentrations for use as standard reference metabolites in targeted metabolomics. The solutions were stored at -80 ℃. Added 300 μL of ice-cold DW containing acetonitrile (1:1, v/v) to each freeze-dried fecal/serum sample. The samples were then ground for 3 min at 60 Hz (-20 ℃ pre-cooling, WONBIO, Shanghai, CHN). After that, ultrasonic extraction was performed for 10 min in ice-water (FUYANG, Shenzhen, CHN). The samples were then centrifuged at 4 ℃ for 10 min at 12,000 rpm (Lu Xiangyi, Shanghai, CHN) to obtain the supernatant. The resulting supernatant was diluted with DW containing acetonitrile (1:1, v/v) to a volume that is 5 times the original volume.

The 80 μL sample/standard reference metabolites solution, 40 μL 200 mM 3-nitrophenylhydrazine and 40 μL 120 mM 1-(3-dimethylaminopropyl)-3-ethylcarbodiimide hydro chloride-6% pyridine (both two were prepared with water containing acetonitrile, 1:1, v/v) were mixed into injection vial. The resulting mixture was then subjected to a 30-min reaction at 40 ℃ (KEXI, Shanghai, CHN), followed by a 1-min incubation on ice. Next, 160 μL of the reacted mixture was extracted using a syringe and filtered through a 0.22 μm organic phase pinhole filter for subsequent UPLC-MS/MS analysis.

Liquid chromatography (LC) analysis was conducted using a Nexera UHPLC LC-30A system (Shimadzu, TKY, JPN) equipped with an ACQUITY UPLC BEH C18 column (1.7 μm, 100 × 2.1 mm, Waters, MA, USA). The injection volume was set at 1 μL, and the flow rate was maintained at 0.4 mL/min. Mobile phase A was composed of 0.1% formic acid (ThermoFisher, MA, USA) in water, while mobile phase B consisted of a mixture of acetonitrile and methanol (2:1, v/v). The following gradient elution program was employed: 0 min, A/B (80:20, v/v); 8 min, A/B (60:40, v/v); 8.1 min, A/B (5:95, v/v); 9.6 min, A/B (80:20, v/v); 10 min, A/B (80:20, v/v). Samples were kept at 4 ℃ throughout the elution process, and the column temperature was maintained at 40 ℃.

MS analysis was performed in the negative ion mode using the ABSCIEX Selex ION Triple Quad^™^ 5500 System (SCIEX, MA, USA), with the electrospray ionization source set at -4500 V and 450 ℃. Nitrogen was employed as the collision gas, and the column temperature was kept at 40 ℃, the flow rate was set as 30 psi. The targeted SCFAs were analyzed utilizing multiple reaction monitoring mode. Data acquisition and analysis were performed using Analyst software (SCIEX, MA, USA), with quantification of all targeted SCFAs conducted using ABSCIEX OS-MQ (SCIEX, MA, USA).

### Cell culture

QGP-1 cells (enterochromaffin cells model) and H9 cells (T cells) were acquired from American Type Culture Collection (ATCC, USA, https://www.atcc.org). Cells were cultured in a 37 ℃ incubator with 5% CO_2_ using RPMI 1640 culture medium (Sigma-Aldrich, MO, USA), supplemented with 10% fetal bovine serum (FBS, Sigma-Aldrich, MO, USA) and 1% penicillin/streptomycin (Sigma-Aldrich, MO, USA). L-tryptophan (L-Trp, Yuanye, Shanghai, CHN) was added at the concentration of 40 μmol/L, and sodium butyrate (Sigma-Aldrich, MO, USA) was added at concentrations of 2 and 4 mmol/L. The antagonist of G protein-coupled receptor 43 (GPR43), GLPG0974 (MedChemExpress, NJ, USA), was used at the concentration of 5 μmol/L, while the agonist of GPR43, TUG-1375 (MedChemExpress, NJ, USA) was used at the concentration of 16 μmol/L. Additionally, 5-hydroxytryptamine (5-HT, Sigma-Aldrich, MO, USA) was supplied at the concentration of 100 μmol/L, the antagonist of 5-HT receptor 1A (HTR1A), Way100635 (MedChemExpress, NJ, USA), was used at the concentration of 10 μmol/L, and the antagonist of 5-HT receptor 3 (HTR3), Tropisetron (Selleck, TX, USA), was used at the concentration of 25 μmol/L. All the supernatant and the cells should be collected for western blot (WB) and real time-quantitative polymerase chain reaction (RT-qPCR) after 48 h.

### Enzyme linked immunosorbent assay (ELISA) and WB

The faeces and grated colonic tissue diluted with DW and centrifuged at 3000 rpm for 30 min, then the supernatant was collected for ELISA. The serum and supernatant from the cell culture were also collected for ELISA. Proteins were extracted from tissue/cell samples using radio immunoprecipitation assay (RIPA, Servicebio, Beijing, CHN), and the protein content was quantified using the PierceTM BCA Protein Assay kit (ThermoFisher, MA, USA).

ELISA was performed according to the manufacturer's instructions for the following cytokines: interleukin-1 beta (IL-1β, Proteintech, Wuhan, CHN), interleukin-2 (IL-2, Proteintech, Wuhan, CHN), interleukin-6 (IL-6, Proteintech, Wuhan, CHN), tumor necrosis factor-alpha (TNF-α, Proteintech, Wuhan, CHN), interferon-gamma (IFN-γ, Proteintech, Wuhan, CHN), and 5-HT (Cloud-Clone, Wuhan, CHN).

The proteins were separated using 12% sodium dodecyl sulfate-polyacrylamide gel electrophoresis (SDS-PAGE, Epizyme, Shanghai, CHN) and subsequently transferred to polyvinylidene fluoride (PVDF) membranes (Servicebio, Wuhan, CHN). The membranes were then incubated with protein-free rapid blocking buffer (1 × , Epizyme, Shanghai, CHN) for 1 h at room temperature, and incubated overnight at 4 ℃ with the relevant primary antibodies: tryptophan hydroxylase 1 (TPH1, ABclonal, Wuhan, CHN), dopa decarboxylase (DDC, Abcam, MA, USA), monoamine oxidase B (MAOB, Proteintech, Wuhan, CHN), aldehyde dehydrogenase 1 family member B1 (ALDH1B1, Proteintech, Wuhan, CHN), sodium-dependent serotonin transporter (SLC6A4, Abcam, MA, USA), GPR43 (Proteintech, Wuhan, CHN), protein kinase C theta (PKCθ, Abcam, MA, USA), p-PKCθ (Bioss, Beijing, CHN), NF-κB (ABclonal, Wuhan, CHN), p-NF-κB (Abcam, MA, USA), HTR1A (Abcam, MA, USA), HTR3 (Abcam, MA, USA) and glyceraldehyde-3-phosphate dehydrogenase (GAPDH, ABclonal, Wuhan, CHN). The membranes were then washed with tris buffered saline with tween (TBST, Servicebio, Wuhan, CHN), and subsequently incubated with HRP-labeled secondary antibodies (ABclonal, Wuhan, CHN) at room temperature for 1 h. Following the wash step, the membranes were exposed using Tanon 4800 (Tanon, Shanghai, CHN) and analyzed using ImageJ (V1.53, https://imagej.nih.gov/ij/, National Institutes of Health, MD, USA).

### RT-qPCR and PCR

Total RNA was extracted from colonic tissue and cell samples using the RNA Extraction kit (Accurate Biology, Changsha, CHN), followed by cDNA synthesis using the Evo M-MLV Reverse Transcription kit (Accurate Biology, Changsha, CHN). Amplification of cDNA was performed using the Maxima SYBR Green qPCR Master Mix kit (ThermoFisher, MA, USA) and RT-qPCR analysis was carried out on the BIO-RAD CFX96 Real-Time PCR System (BIO-RAD, CA, USA) and Applied Biosystems ViiA^™^ 7 Real-Time PCR System (ThermoFisher, MA, USA), following the manufacturer's protocols. Total DNA was extracted from the digesta in the colon using the QIAamp PowerFecal Pro DNA kit (QIAGEN, Dusseldorf, GER), and PCR analysis was performed on the Applied Biosystems ViiA™ 7 Real-Time PCR System (ThermoFisher, MA, USA).

The primers were as follows: Mouse-Gapdh (5’-CCATGGAGAAGGCTGGGG-3’, 5’-CAAAGTTGTCATGGATGACC-3’), Human-GAPDH (5’-GGAGCGAGATCCCTCCAAAAT-3’, 5’-GGCTGTTGTCATACTTCTCATGG-3’), Human-HTR1A (5’-AAGACAGTGAAGACGCTGGG-3’, 5’-AGAAGGGCAGAACAAGAGCC-3’), Human-HTR3 (5’-GCAGGAAAACTTGGCAGCTC-3’, 5’-CTCTGCCAACCTCATGTCCC-3’), Mouse-Tph1 (5’-CAGTGGCTCTGAGGTGAGTG-3’, 5’-AGTTGATCCCGTCCTTGCTG-3’), Human-TPH1 (5’-TCTACCCAACCCATGCTTGC-3’, 5’-AAGTAACCAGCCACAGGACG-3’), Mouse-Ddc (5’-GAGCTGGACAATCCCGACAA-3’, 5’-GATCAGGGGCCGAAGATAGC-3’), Human-DDC (5’-ACTCCCGGCTGCCTTTTTC-3’, 5’-CGTCCCTCAATGCCTTCCAT-3’), Mouse-Maob (5’-CCACATTGACCAGACAGGGG-3’, 5’-TCTTCATGCCCAAAGCAGGT-3’), Human-MAOB (5’-AGCGCCATGAGCAACAAATG-3’, 5’-AGGATCCTCCAAGGTCCACA-3’), Mouse-Aldh1b1 (5’-TGCTGAACTGACCGGAGAAC-3’, 5’-TTTGGGATTGGGTTCGGGAG-3’), Human-ALDH1B1 (5’-AGCCTGCTGCAGAGTGTCAG-3’, 5’-CTTGCTGACTGCATCTTGCC-3’), Mouse-Slc6a4 (5’-CAAAACGTCTGGCAAGGTGG-3’, 5’-ACACCCCTGTCTCCAAGAGT-3’), Human-SLC6A4 (5’-TGCAGAAGCGATAGCCAACA-3’, 5’-GTGGGAACTCATCCAGCACA-3’), Mouse-Gpr43 (5’-TGACAGGCTTCGGCTTCTACAG-3’, 5’-AGAGCAGCGATCACTCCATACAG-3’), Human-GPR43 (5’-GAGCAGGTCAGAAGTGGCAATG-3’, 5’-GAAGAAGAGCACCAGGCACAG-3’), Human-PKCθ (5’-CGAGTTCACTGCCACCTTCTTC-3’, 5’-GCACTGGTAGCCCTGTTTGTTC-3’), Human-NF-κB (5’-GCCCACTCGCTGCCTCTC-3’, 5’-ATGTCTCCACGCCGCTGTC-3’).

### Hematoxylin–eosin (HE) staining, immunohistochemistry (IHC) and immunofluorescence (IF)

Following sacrifice and dissection, the colon of each mouse was promptly extracted and perfused with normal saline. The colons of mice from different groups were fixed in 4% paraformaldehyde for 24 h, subjected to dehydration in gradient ethanol, and subsequently embedded in paraffin. The paraffin-embedded tissue was sectioned into slices of 5 μm thickness, followed by HE staining to evaluate histopathology using an inverted microscope (Leica Microsystems, Wetzlar, Germany).

Rabbit primary antibodies targeting 5-HT (Abcam, MA, USA) were selected for IHC. Secondary antibodies, derived from goats, were incubated with the samples after which diaminobenzidine (DAB, ZSGB-BIO, Beijing, CHN) was used for signal visualization, and the sections were counterstained with hematoxylin. Finally, the target proteins were observed using an inverted microscope and analyzed using ImageJ.

Sections were washed 3 times with PBST (phosphate buffered saline with tween, Servicebio, Beijing, CHN) and then blocked in 10% normal goat serum, 5% bovine serum albumin (BSA, Servicebio, Beijing, CHN) and 0.3% Triton X-100 (Servicebio, Beijing, CHN) diluted in PBST at room temperature for 1 h. Firstly, antibodies of CD4 (Abcam, MA, USA) was incubated overnight at 4 ℃, and then antibody of PKCθ was incubated for 1 h at room temperature. Nucleus were stained with 4',6-diamidino-2-phenylindole (DAPI, Solarbio, Beijing, CHN). IF images were captured using a Leica TCS SP8 confocal microscope (Leica Microsystems, Wetzlar, Germany).

### Statistical analysis

Statistical analysis was performed using the Statistical Package for Social Sciences (SPSS) software (V25.0, SPSS Inc., IL, USA). The normal distribution of variables was assessed using the Shapiro-Wilk test. Normally distributed variables were analyzed using the one-way analysis of variance (ANOVA) procedure, while non-normally distributed variables were assessed using the Mann-Whitney U test. Statistical significance was determined at *P* ≤ 0.05.

## Results

### SYD alleviated the colonic inflammation in DSS-induced mice

**Fig. 1:**
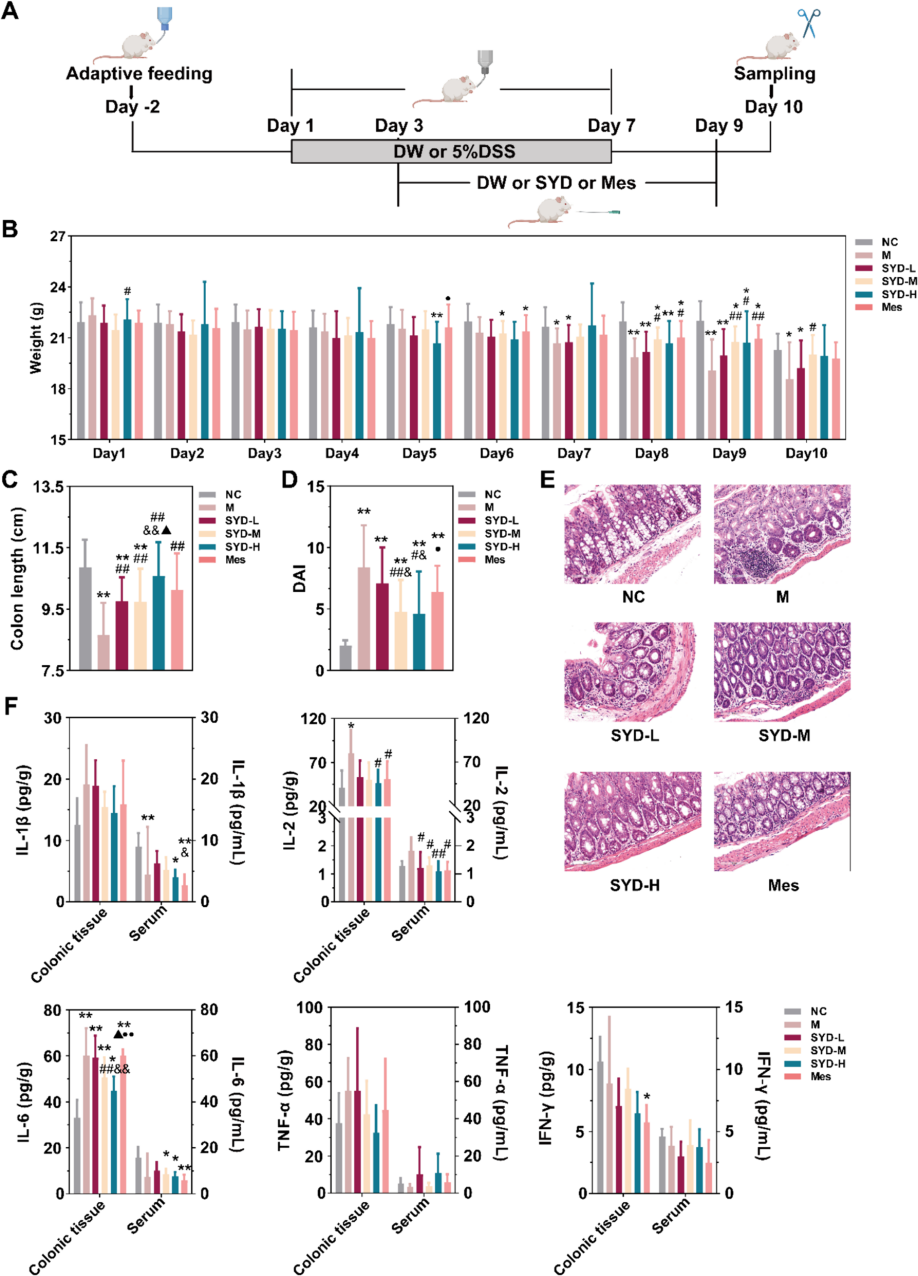
SYD alleviated the colonic inflammation in DSS-induced mice. **A** Experimental schedule. Changes of **B** weight, **C** colon length, and **D** the DAI scores during the experiment. DAI = weight-losing score + bloody stool score + stool shape score [15]; weight-losing score: 0 - no weight-losing, 1 - weight-losing between 1 and 5%, 2 - weight-losing between 5 and 10%, 3 - weight-losing between 10 and 15%, 4 - weight-losing more than 15%; blood stool score: 0 - no bloody stool, 2 - positive occult blood test without bloody stool in naked eye, 4 - blood stool in naked eye; stool shape score: 0 - normal shape, 2 - soft strip stool, 4 - loose stool. **E** Representative images of HE stained sections of the colon (20 × , 100 μm). **F** The concentrations of IL-1β, IL-2, IL-6, TNF-α and IFN-γ in the colonic tissue and the serum. Compared with NC, ^*^P ≤ 0.05, ^**^P ≤ 0.01; compared with M, ^#^*P* ≤ 0.05, ^##^*P* ≤ 0.01; compared with SYD-L, ^&^*P* ≤ 0.05, ^&&^*P* ≤ 0.01; compared with SYD-M, ^▲^*P* ≤ 0.05, ^▲▲^*P* ≤ 0.01; compared with SYD-H, ^●^*P* ≤ 0.05, ^●●^*P* ≤ 0.01

As shown in Fig. 1A, DSS-induced mice were orally administered with SYD or mesalazine upon the observation of diarrhea and positive occult blood tests in over half of the mice. DW was given to the normal control (NC) group. Serum, fecal (digesta in the colon) and colonic tissue samples were collected for HE staining and ELISA analysis to assess the therapeutic effect of SYD in mice. The DSS-induced mice presented significant weight loss, colon shortening and an increased disease activity index (DAI) (Fig. 1B-D). Microscopic examination of HE stained samples showed features of inflammatory infiltration, crypt abscesses and reduced goblet cells (Fig. 1E). However, both SYD and mesalazine significantly mitigated these damages, with a dose-dependent effect observed specifically in the SYD treated groups (Fig. 1C-E). Notably, SYD treatment did not result in an improvement of the thymus index, despite the reduction observed in DSS-induced mice (Supplementary Fig. 1A). In contrast, the spleen index significantly increased following SYD treatment (Supplementary Fig. 1A). Furthermore, the levels of cytokines in the colonic tissue and serum showed a significant improvement after SYD treatment, the levels of IL-2 and IL-6 were initially elevated in DSS-induced mice but demonstrated dose-dependent decreases in mice treated with SYD (Fig. 1F), as well as the expression of NF-κB (Supplementary Fig. 1B).

### The pharmacology mechanism of SYD based on the ingredients identified in UPLC-MS/MS and the targeted genes screened out in TCMSP

The positive and negative ion UPLC-MS/MS analysis of SYD identified paeoniflorin, baicalin and berberine as the top three peaks (Supplementary Fig. 2A-H), and the quantifications of these three key ingredients were further performed (Supplementary Fig. 2I). Out of the 35 ingredients in SYD, the TCMSP database revealed 92 potential targeted genes (Supplementary Table 1 & 2). Additionally, the enrichment analysis based on these targeted genes identified 33 PANTHER pathways, including those associated with infection, apoptosis, nutrition, immunity and inflammation (Supplementary Fig. 2J). Notably, significant enrichment was observed in pathways related to 5-HT, such as 5-HT degredation and 5HT2 receptor-mediated signaling pathway. Key targeted genes involved in these pathways included monoamine oxidase A (MAOA), MAOB, SLC6A4 and 5-HT receptor 2A (HTR2A) (Supplementary Table 2).

### SYD regulated the 5-HT metabolism and gene expression in DSS-induced mice

**Fig. 2:**
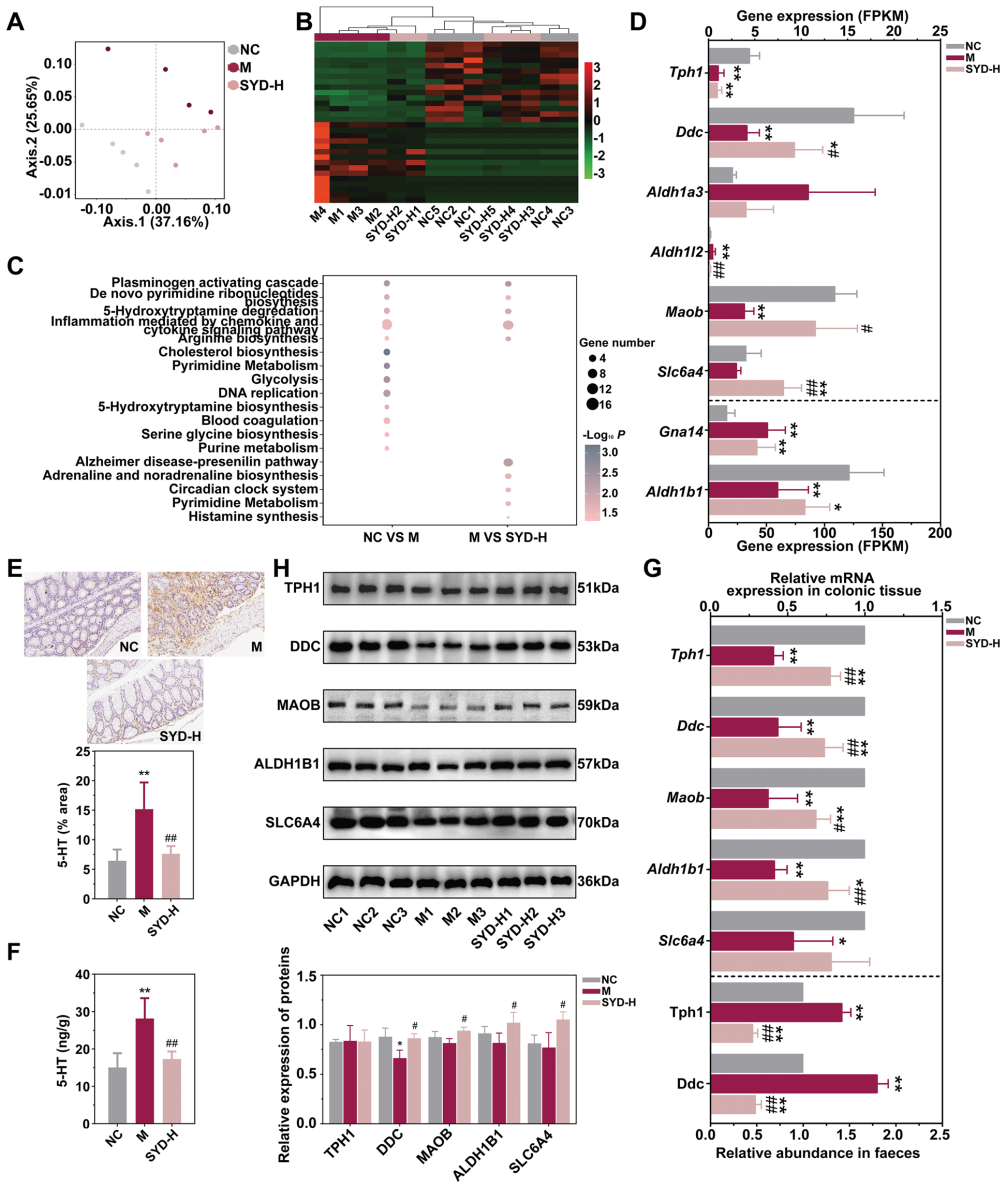
SYD regulated the gene expression in the colon from DSS-induced mice. **A** PCoA based on all gene expression profile (Adonis: R^2^ = 0.396, *P* = 0.000). **B** Heatmap revealed the differences of gene expression (top 15 up-regulated DEGs and top 15 down-regulated DEGs by SYD). **C** Core PANTHER pathways that played important roles in SYD treatment. **D** The expressions of genes related to 5-HT metabolism in mRNA sequencing. **E** IHC of the colonic tissue with 5-HT antibody. Scale bars, 20 × , 100 μm. **F** The 5-HT concentrations in the faeces. **G** The expressions of genes related to 5-HT metabolism in the colonic tissue and the relative abundance of genes about 5-HT synthesis in the faeces. **H** The expressions of proteins related to 5-HT metabolism in the colon that confirmed in WB. Compared with NC, ^*^P ≤ 0.05, ^**^P ≤ 0.01; compared with M, ^#^*P* ≤ 0.05, ^##^*P* ≤ 0.01

We performed gene expression analysis in the colonic tissues of DSS-induced mice and SYD treated mice (Supplementary Table 3). The gene expression profiles showed marked differences between the DSS-induced and NC mice, as shown by the distinct clustering in the PCoA and the most DEGs among groups (Fig. 2A: Adonis: R^2^ = 0.396, *P* = 0.000, Supplementary Fig. 3A-C: Adonis: R^2^ = 0.499, *P* = 0.000, Supplementary Table 4-8). Treating mice with SYD and Mes resulted in the reversal of these gene expression changes, as evidenced by the gene expression profiles of the SYD-H and Mes groups resembling those of the NC mice and exhibiting a larger number of DEGs compared to the DSS-induced mice (Fig. 2A, B, Supplementary Fig. 3A, Supplementary Table 7 & 8). Additionally, PANTHER and KEGG pathway enrichment analysis were performed to investigate the mechanisms underlying the actions of SYD based on the DEGs (Supplementary Fig. 3D). We identified 18 core pathways in PANTHER classification system, including pathways related to 5-HT biosynthesis and degredation (Fig. 2C), as well as 37 core pathways in KEGG database, including tryptophan metabolism (Supplementary Fig. 3E), which played significant roles in 5-HT metabolism. Based on these pathways, we summarized the key genes involved in 5-HT biosynthesis and degredation, as shown in Supplementary Fig. 3F, including *Tph1*, *Ddc*, *Aldh1b1* and *Maob*. Notably, SYD could reverse the significant reduction of *Maob* in DSS-induced mice (Fig. 2D), which might result in the decreasing concentrations of 5-HT in colonic tissue and feaces (Fig. 2E, F).

These findings support the idea that 5-HT metabolism is a specific pathway targeted by SYD treatment, which was consistent with the previous study [14]. However, the treatment with SYD resulted in the up-regulation of key enzymes involved in 5-HT degredation (ALDH1B1 and MAOB) in the colonic tissue of DSS-induced mice (Fig. 2D, G, and H), and down-regulated the key enzymes involved in 5-HT biosynthesis (Tph1 and Ddc) in the faeces of DSS-induced mice (Fig. 2G), even though these two exhibited contrasting changes in the colonic tissue (Fig. 2D, G, and H). These results suggest that SYD modulates 5-HT metabolism by regulating both the biosynthesis and degredation pathways, potentially involving the intestinal flora in the faeces.

### The involvement of SYD in 5-HT metabolism via intestinal flora and its metabolites

**Fig. 3:**
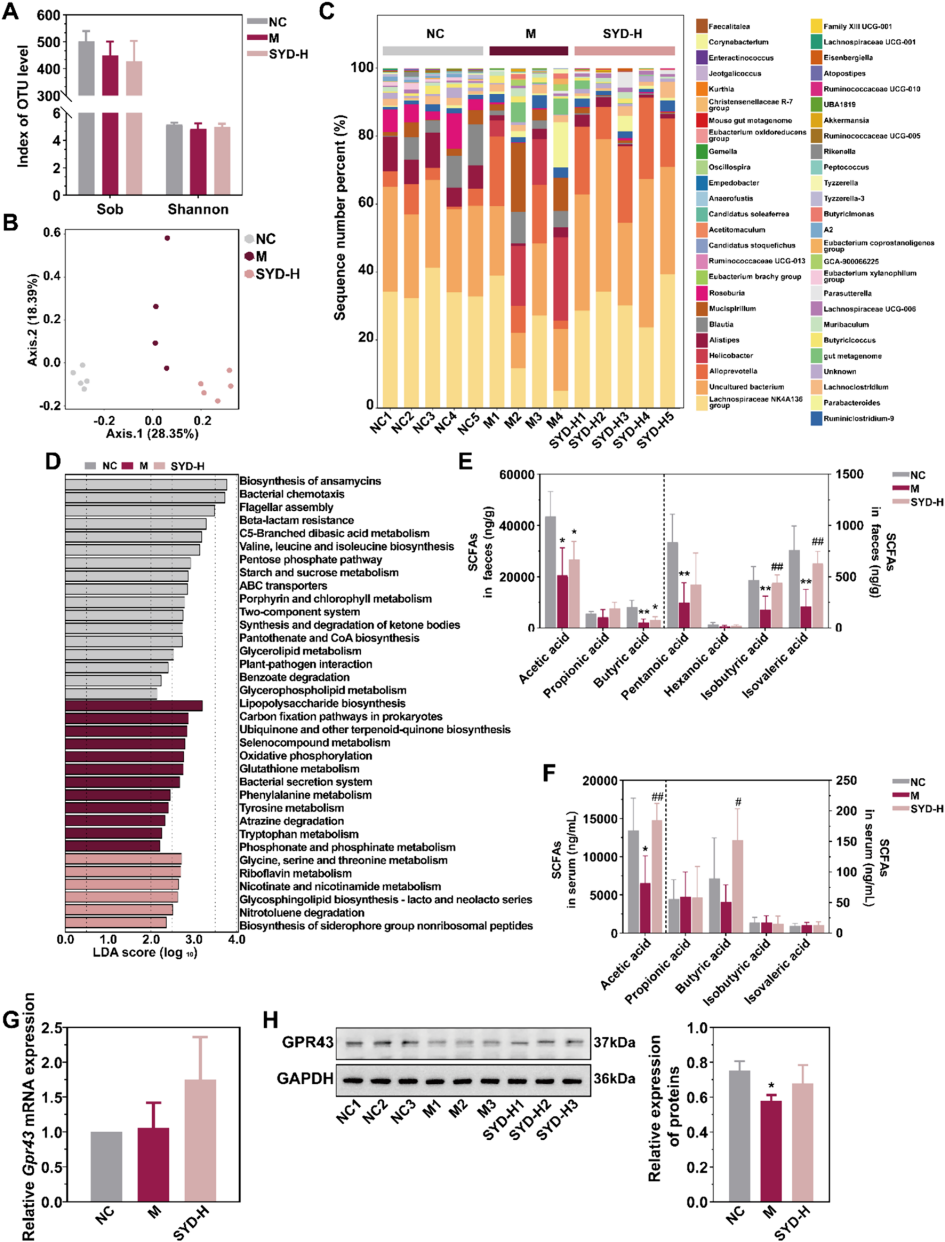
SYD regulated the SCFAs metabolism from the intestinal flora. **A** Analysis of the bacterial diversity - Sobs index and Shannon index. **B** PCoA based on Bray-Curtis distance (Adonis: R^2^ = 0.458, *P* = 0.000). **C** Relative abundances of the top 50 genera. **D** LEfSe analysis of the KEGG pathways based on the predicted 16S rDNA genes by PICRUSt algorithm (LDA score ≥ 2 and *P* ≤ 0.05). SYD both promoted SCFAs production in the **E** faeces and **F** serum. **G** Expression of the *Gpr43* mRNA in the colonic tissues confirmed by RT-qPCR. **H** Expression of GPR43 determined by WB analysis and normalized to GAPDH. 3 mice per group. Compared with NC, ^*^P ≤ 0.05, ^**^P ≤ 0.01; compared with M, ^#^*P* ≤ 0.05, ^##^*P* ≤ 0.01

It was no doubt that approximately 90% of 5-HT was generated under the participation of intestinal flora, and our results also prompted that the intestinal flora coded the genes related to 5-HT biosynthesis (Tph1 and Ddc, which were both down-regulated by SYD, Fig. 2G). However, further investigation is needed to understand the specific mechanism by which the intestinal flora influences the expression of genes related to 5-HT in the organism. Consequently, we evaluated the composition of the intestinal flora by sequencing 16S rDNA in this study. Nevertheless, there were no notable variations in community richness and diversity among the NC and DSS-induced mice, as well as between the DSS-induced mice and those treated with SYD, as indicated by the Sob, Shannon, Ace, and Chao indexes (Fig. 3A, Supplementary Fig. 4A, Supplementary Table 9). The Kruskal-Wallis test of beta distances revealed a substantial rise in mice exposed to DSS, but no significant distinction was found between the DSS-induced mice and those treated with SYD or Mes (Supplementary Table 10, Supplementary Fig. 4B, C, Fig. 3B: Adonis: R^2^ = 0.458, *P* = 0.000). Unlike the similar proportions of most of the top 50 genera, specific dominant genera displayed notable variations in distribution between NC mice and DSS-induced mice, as well as between DSS-induced mice and those treated with SYD, including *Lachnospiraceae NK4A136 group* and *Oscillospira*, which are known for their involvement in butyric acid production (Fig. 3C, Supplementary Fig. 4D, Supplementary Table 10 & 11). The changes in their abundance provided confirmation of the anticipated impact of SYD treatment. Furthermore, an enrichment analysis of the KEGG pathway was conducted using the predicted 16S rDNA genes (Supplementary Table 12). LEfSe analysis demonstrated the enrichment of the pathway related to tryptophan metabolism, which plays a regulatory role in 5-HT synthesis, in the DSS-induced mice (Fig. 3D). These findings aligned with the alterations detected in Tph1 and Ddc levels in the faeces (Fig. 2G) and the concurrent changes in 5-HT concentrations in the colonic tissues, faeces and serum (Fig. 2E, F) [14].

In addition, the levels of SCFAs were measured in both the faeces and serum. The results revealed a negative correlation between 5-HT concentrations and butyric acid in the faeces, as well as isobutyric acid and isovaleric acid in the faeces (Fig. 3E, F, Supplementary Fig. 5). The presence of higher levels of the latter two SCFAs in the faeces could be indicative of their involvement in 5-HT metabolism within the intestinal flora, whereas the high concentration of butyric acid in the serum may play a role in regulating 5-HT metabolism in the organism. Previous studies have shown that SCFAs, through GPR43, can hinder TPH1 transcription in enterochromaffin cells (ECCs) [16], while L-Trp can be converted to 5-HT by hydroxylation and decarboxylation in the presence of TPH1. Our study observed an increase in GPR43 expression in DSS-induced mice treated with SYD-H, although this did not reach statistical significance (Fig. 3G, H).

### Sodium butyrate regulated 5-HT metabolism in QGP-1 cells by inhibiting 5-HT synthesis and promoting its degredation

Besides instigating local inflammation in the colon, DSS induced an up-regulation in the relative abundances of genes associated with 5-HT synthesis in the intestinal flora (Fig. 2G), thereby partially contributing to the elevated 5-HT concentration in the colon (Fig. 2E, F) [14]. Conversely, DSS hindered the colonization of bacteria capable of producing butyric acid, including *Lachnospiraceae NK4A136 group* and *Oscillospira* (Fig. 3C, Supplementary Fig. 4D, Supplementary Table 10 & 11), and the resulting metabolite, decreasing butyric acid, has the potential to influence 5-HT metabolism in the organism (Fig. 3E-H, Supplementary Fig. 5), thus making the intestinal flora/butyric acid/5-HT metabolism as a prospective target for SYD. The specific mechanism will be further investigated utilizing the QGP-1 cells (ECC model).

**Fig. 4:**
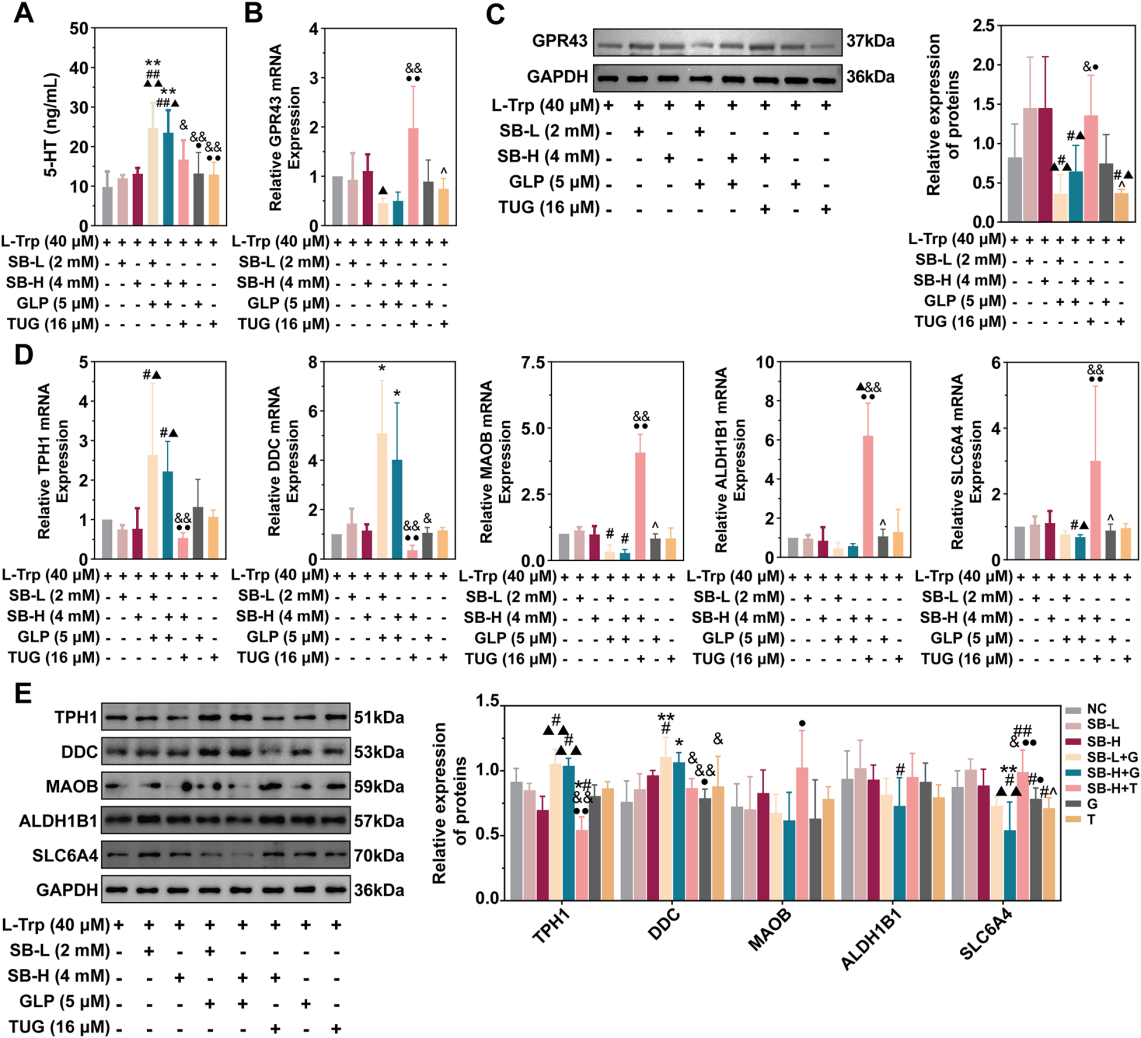
Sodium butyrate regulated 5-HT metabolism through GPR43 in QGP-1 cells (ECC model). **A** Sodium butyrate and the GPR43 inhibitor/activator regulated the synthesis of 5-HT in QGP-1 cells. **B**-**C** Sodium butyrate and the GPR43 inhibitor/activator intervened the expression of GPR43 in QGP-1 cells. **D**-**E** Sodium butyrate and the GPR43 inhibitor/activator regulated the expressions of key enzymes in 5-HT metabolism in QGP-1 cells. GLP, GLPG0974; TUG, TUG-1375. NC, L-tryptophan (40 μM) treatment; SB-L, sodium butyrate (2 mM) and L-Trp (40 μM) treatment; SB-H, sodium butyrate (4 mM) and L-Trp (40 μM) treatment; SB-L + G, sodium butyrate (2 mM), GLPG0974 (5 μM) and L-Trp (40 μM) treatment; SB-H + G, sodium butyrate (4 mM), GLPG0974 (5 μM) and L-Trp (40 μM) treatment; SB-H + T, sodium butyrate (4 mM), TUG-1375 (16 μM) and L-Trp (40 μM) treatment; G, GLPG0974 (5 μM) and L-Trp (40 μM) treatment; T, TUG-1375 (16 μM) and L-Trp (40 μM) treatment. Compared with NC, ^*^P ≤ 0.05, ^**^P ≤ 0.01; compared with SB-L, ^#^*P* ≤ 0.05, ^##^*P* ≤ 0.01; compared with SB-H, ^▲^*P* ≤ 0.05, ^▲▲^*P* ≤ 0.01; compared with SB-L + G, ^&^*P* ≤ 0.05, ^&&^*P* ≤ 0.01; compared with SB-H + G, ^●^*P* ≤ 0.05, ^●●^*P* ≤ 0.01; compared with SB-H + T, ^^^*P* ≤ 0.05

High dose of L-Trp significantly reduced the viability of QGP-1 cells, which can be reversed by low dose of sodium butyrate. Consistently, GPR43 inhibitor (GLPG0974) can decrease the viability of QGP-1 cells, while low dose of GPR43 activator (TUG-1375) can increase the viability (Supplementary Fig. 6A). However, the addition of high dose of sodium butyrate did not significantly reduce the concentration of 5-HT in the culture supernatant (Fig. 4A). Comparing with sodium butyrate, the inhibitor of GPR43 increased 5-HT concentrations, and the activator of GPR43 displayed insignificant inhibition on 5-HT synthesis, but with dramatic decrease in 5-HT concentration comparing with the inhibitor (Fig. 4A). These results are contrary to the expression of GPR43 and suggest that GPR43 suppresses the synthesis of 5-HT (Fig. 4B, C). Consistently, the downstream genes about 5-HT metabolism also exhibited the relevant changes, when treating with the inhibitor of GPR43, the expressions of genes/enzymes about 5-HT synthesis (TPH1 and DDC) were up-regulated while the genes/enzymes about 5-HT degredation/transport (MAOB, ALDH1B1 and SLC6A4) were down-regulated, which were all reversed by the activator of GPR43 (Fig. 4D, E), indicating that GPR43 is the target of sodium butyrate in regulating 5-HT metabolism in the organism.

### 5-HT promoted the activation of T lymphocyte and regulated the secretion of cytokines via HTR1A and HTR3

**Fig. 5:**
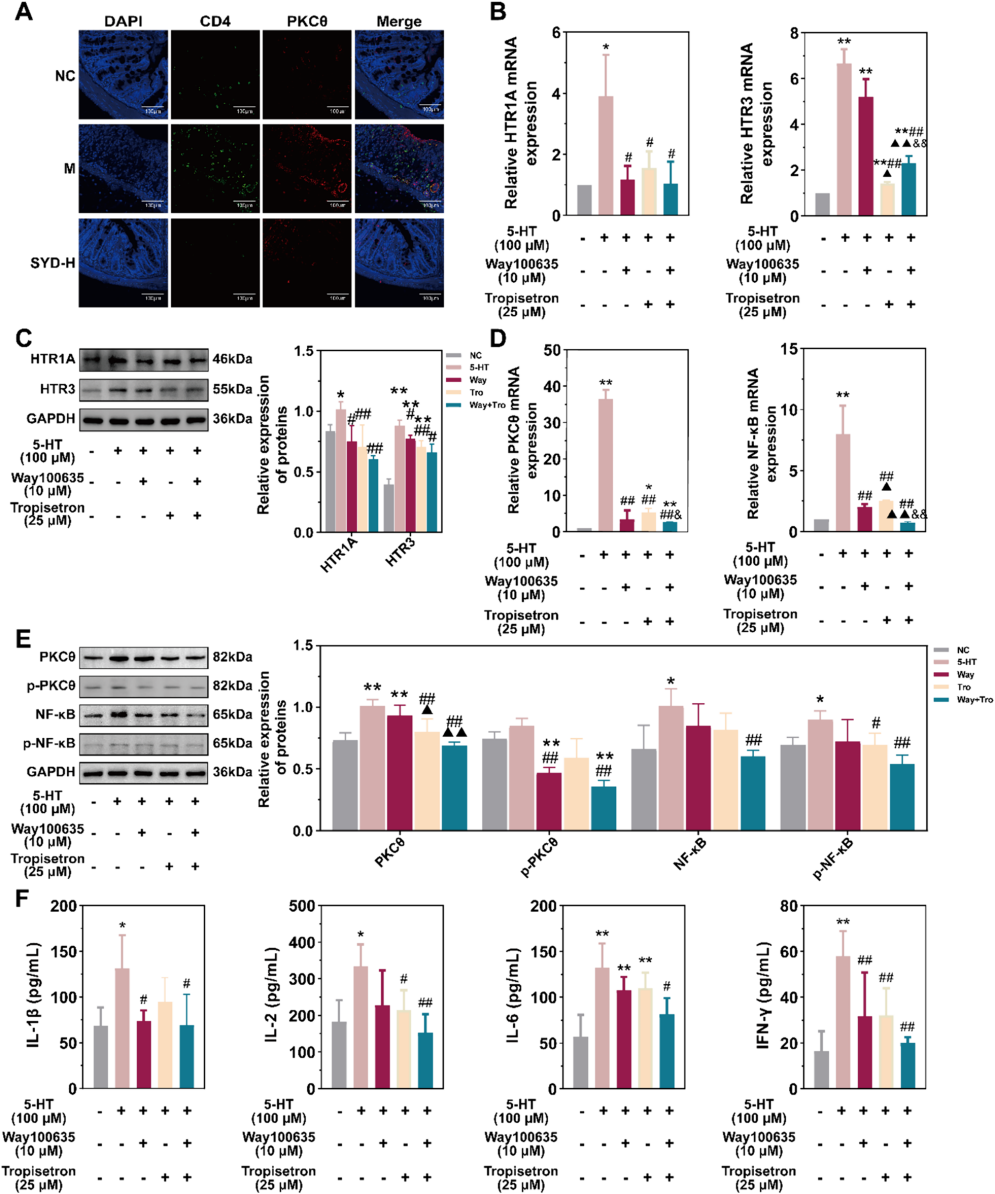
5-HT promoted the activation of H9 cells (T lymphocyte line). **A** IF of inflammatory proteins PKCθ in CD4^+^ T lymphocytes in the colonic tissue from mice experiments. CD4: green; PKCθ: red; nucleus: blue. Scale bars = 100 µm. **B**-**C** 5-HT regulated the expression of HTRs in H9 cells. **D**-**F** 5-HT promoted the inflammation in H9 cells. NC, negative control; 5-HT: 5-HT (10 μM) treatment; Way: 5-HT (10 μM) and Way100635 (10 μM) treatment; Tro: 5-HT (10 μM) and Tropisetron (25 μM) treatment; Way + Tro: 5-HT (10 μM), Way100635 (10 μM) and Tropisetron (25 μM) treatment. Compared with NC, ^*^P ≤ 0.05, ^**^P ≤ 0.01; compared with 5-HT, ^#^*P* ≤ 0.05, ^##^*P* ≤ 0.01; compared with Way, ^▲^*P* ≤ 0.05, ^▲▲^*P* ≤ 0.01; compared with Tro, ^&^*P* ≤ 0.05, ^&&^*P* ≤ 0.01

The above results indicate that SYD reduces the levels of 5-HT in the colon by directly and indirectly inhibiting its synthesis and promoting its degredation. It has indicated that PKCθ plays a central role in T lymphocyte activation and survival in previous studies [17, 18], thus we further employed IF in the colonic tissues from DSS-induced and SYD-treated mice to explore the status of T lymphocytes and the expression of PKCθ in colitis. As shown in Fig. 5A, CD4^+^ T lymphocyte infiltrated in the colon of DSS-induced mice, that indicated the immune disorders in colitis, and that could be improved by SYD dramatically. Moreover, the up-regulated PKCθ expressed in T lymphocyte, were also reduced by SYD markedly (Fig. 5A). Therefore, we supposed that T lymphocyte was the downstream target of 5-HT and conducted the subsequent experiments using H9 cells. As shown in Supplementary Fig. 6B, 5-HT and its receptor inhibitors (Way100635 - inhibitor of HTR1A, Tropisetron - inhibitor of HTR3) were added to the H9 cell culture at appropriate concentrations. As expected, the inhibitors noticeably reduced the up-regulation of 5-HTR expression in the presence of 5-HT (Fig. 5B, C), and HTR1A and HTR3 were already determined to be associated with T lymphocyte activation in previous studies [19, 20]. Furthermore, the down-regulation of 5-HTR expression led to a statistically significant decrease in the levels of inflammatory proteins (PKCθ, p-PKCθ, NF-κB and p-NF-κB) and cytokines (IL-1β, IL-2, IL-6 and IFN-γ) induced by 5-HT (Fig. 5D-F). As a result, we conclude that 5-HT in the local colon, as the therapeutic target of SYD, can potentially intervene in colonic inflammation by activating PKC and NF-κB signaling pathways and promoting the secretion of relevant cytokines in T lymphocytes via HTR1A and HTR3.

## Discussion

This study demonstrated that SYD had a dose-dependent effect in alleviating colonic inflammation in DSS-induced mice (Fig. 1). This confirms the effectiveness of this TCM formula that had the specified ingredients from the MS analysis (Supplementary Fig. 2A-H). Through the use of network pharmacology, mRNA sequencing and 16S rDNA sequencing, it was confirmed that SYD directly and indirectly targeted the metabolism of 5-HT (Fig. 2C-F and 3D, Supplementary Fig. 2I & 3E & 3F) [14]. The key enzymes involved in the synthesis, transport and degredation of 5-HT were detected in DSS-induced mice and SYD treated mice. SYD directly reduced the relative abundance of Tph1 and Ddc in the intestinal flora (Fig. 2G), resulting in a decreased concentration of 5-HT in the local colon (Fig. 2E, F). This indicated that SYD can directly regulate the 5-HT synthesis in the intestinal flora. However, the expression of TPH1 and DDC in the colonic tissues from mice displayed the contrary changes with Tph1 and Ddc in the feaces (Fig. 2G, H), that might be the negative feedback of the 5-HT concentration and influenced by multi-factors in the organism. Additionally, SYD promoted the production of butyric acid from the intestinal flora (Fig. 3E, F). Although the expression of GPR43, the typical receptor of butyric acid, was influenced by SYD, the change was not statistically significant (Fig. 3G, H). Nevertheless, GPR43 still played a role in the metabolism of 5-HT in the organism. Subsequently, QGP-1 cells were utilized as ECC models to investigate the effects of sodium butyrate on 5-HT metabolism. Sodium butyrate, which is the typical butyrate, althrough didn’t exhibit significant inhibition on the synthesis of 5-HT and promotion on its degredation when comparing with the resting state, its function on the 5-HT metabolism was same to the activator of GPR43 and dramatically blocked by the inhibitor of GPR43 (Fig. 4A-E). This finding suggests that butyric acid intervenes 5-HT metabolism, and provides an explanation for the observed changes in 5-HT concentrations in the local colon during the mice experiment. Interestingly, via HTR1A and HTR3 (Fig. 5B, C), the reduction of 5-HT under the regulation of SYD decreased the activation of PKC and NF-κB signaling pathways in T lymphocytes (Fig. 5D, E), subsequently reducing the secretion of related cytokines (Fig. 5F), ultimately alleviating local colonic inflammation. These results collectively indicate that SYD alleviates UC by inhibiting T lymphocyte activation through 5-HT metabolism, and it targeted multi-targets both in the intestinal flora and the organism.

Previous studies have suggested that 5-HT is synthesized and secreted by ECCs in the organism, involving the intestinal flora and its metabolites [21]. However, our study found an increased relative abundance of Tph1 and Ddc in the faeces in DSS-induced mice, which was subsequently inhibited by SYD (Fig. 2G). These findings indicate that the intestinal flora is involved in 5-HT synthesis, while SYD directly regulates the abundance of these related genes in the intestinal flora. Based on the Spearman’s correlation coefficients between the key differential genera and the relative abundance of Tph1 and Ddc in the faeces (Supplementary Fig. 7), the bacteria that synthesized 5-HT and regulated by SYD, included *Empedobacter*, *Peptococcus*, *Tyzzerella, Helicobacter* et al*.* These genera are commonly associated with inflammation [22, 23]. *Tyzzerella* and *Helicobacter* were reported to be closely related to the intestinal inflammation and increased after the intervention of DSS [24, 25], that was consistent with our results and their genome might contain genes coding TPH1 and DDC. Furthermore, *Tyzzerella* is known for its abundant production of aromatic amines through the action of aromatic amino acid decarboxylase. The product of this enzyme, phenylethylamine, contributes to the peripheral production of 5-HT in the host [26]. *Helicobacter* is recognized for its ability to activate the NF-κB signaling pathway and secretes effector molecules that regulate inflammation, as well as the proliferation and apoptosis of localized cells [27, 28]. On the other hand, it has been confirmed that CagA-positive *Helicobacter* affects the secretion of various hormones, including 5-HT, and these changes can lead to local intestinal inflammation [29, 30].

Additionally, SYD regulated 5-HT metabolism through the metabolites from the intestinal flora, especially the butyric acid-producing bacteria (such as *Lachnospiraceae NK4A136 group* and *Oscillospira*) and their metabolite - butyric acid (Fig. 3C, E, and F) [31]. *Lachnospiraceae NK4A136 group* has the ability to produce SCFAs by fermenting dietary polysaccharides [32, 33]. SCFAs have been found to have an inverse association with various metabolic diseases and chronic inflammation [34, 35]. Meanwhile, SCFAs inhibit histone deacetylases and activate GPRs, two types of receptors found in both intestinal epithelial cells and immune cells [31], to activate (e.g., hypoxia-inducible factor 1, STAT3, and specificity protein 1) or repress (e.g., NF-κB) the transcription factors, then increase the antimicrobial peptides production and suppress the inflammation in the intestinal mucosa [36–38]. Butyric acid, a crucial component of SCFAs, not only has direct anti-inflammatory effects but also serves as the primary energy source for colonocytes, which contributes to the maintenance of intestinal epithelial integrity and prevents local inflammation in the colon [39, 40]. *Lachnospiraceae NK4A136 group* belongs to the Firmicutes phylum, which phylum is widely acknowledged as a significant producer of butyric acid in the human colon and possesses the enzymes butyryl-coenzyme A and acetate-coenzyme A transferase [40, 41]. *Lachnospiraceae NK4A136 group*, as potential producers of butyric acid, also expresses enzymes involved in butyric acid synthesis, such as butyryl-coenzyme A dehydrogenase and butyryl-coenzyme A transferase [41, 42]. *Oscillospira*, a characteristic butyric acid-producing bacterium, is strongly negatively correlated with inflammatory diseases, including UC [43, 44]. By promoting the production of IL-10 from Th1 cells and IL-22 from CD4^+^ T lymphocytes and innate lymphoid cells, butyric acid helps protect the intestine from inflammatory damage [45]. Moreover, oral administration of butyric acid significantly alleviated colonic mucosal inflammation in DSS-induced mice. The mechanism of action includes the activation of leukocytes, regulation of innate immunity and modulation of oxidative stress, with neutrophils possibly being the target [46].

**Fig. 6:**
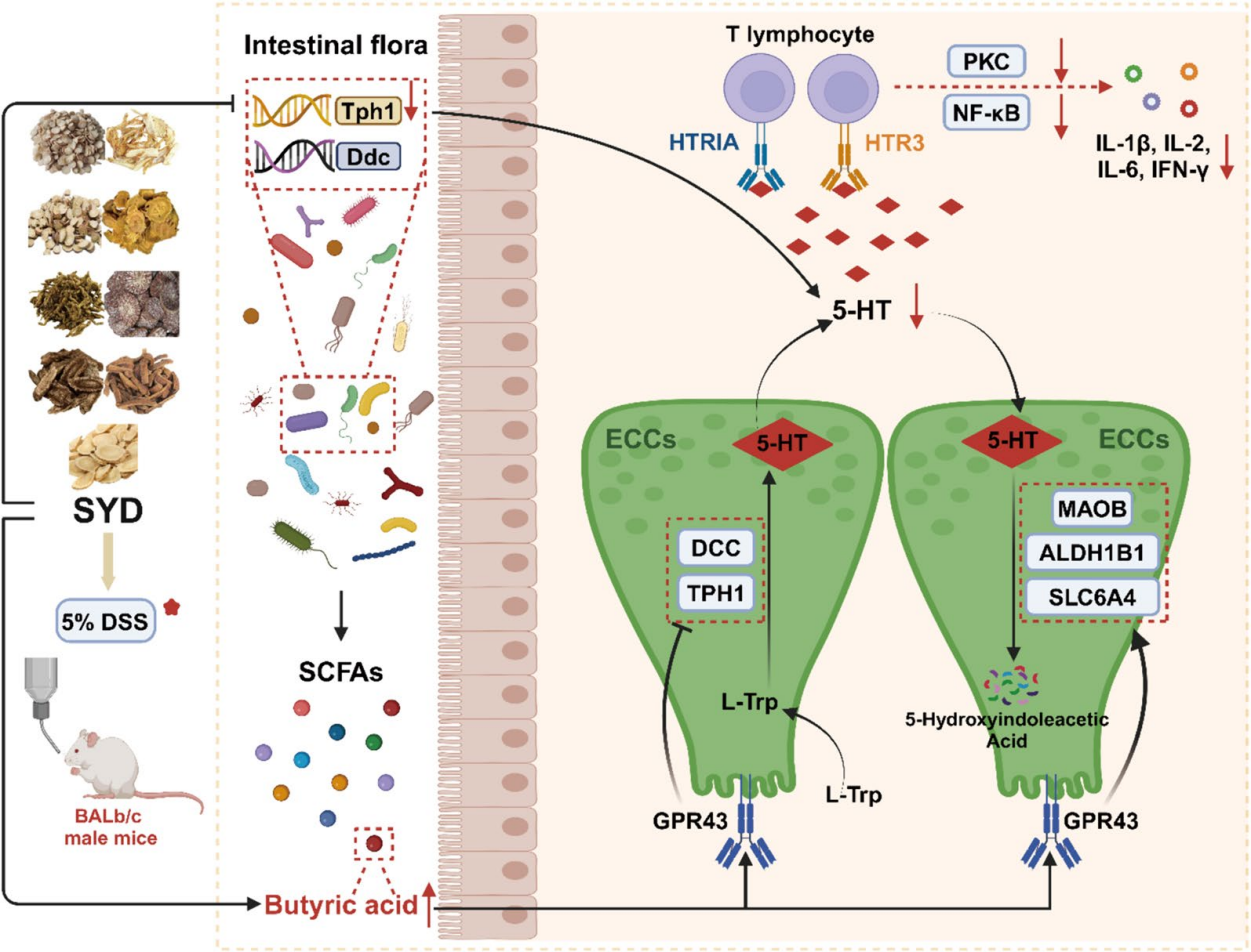
SYD alleviated the colonic inflammation in DSS-induced mice by regulating the T lymphocyte activation caused by 5-HT metabolism in the local colon through the intestinal flora and its metabolites

Besides anti-inflammatory effects, the intestinal flora and its metabolites, such as butyric acid, have been demonstrated to regulate the synthesis of 5-HT in ECCs; however, the precise mechanism remains unclear [47, 48]. ECCs typically synthesize and secrete 5-HT using L-Trp as a substrate [19]. L-Trp undergoes sequential catalysis by TPH1 and DDC to be converted into 5-HT (Fig. 6, Supplementary Fig. 3F). Once activated, 5-HT is transported by SLC6A4 and metabolized by monoamine oxidase in the mitochondrion, thereby affecting its concentration (Fig. 6, Supplementary Fig. 3F) [49, 50]. Although GPR43, a common receptor of butyric acid, is found on ECCs [51], its expression is not significantly affected by sodium butyrate in QGP-1 cells (Fig. 4B, C). This finding is consistent with the animal experiment (Fig. 3G, H). Nonetheless, previous studies have shown that activation of GPR43 inhibits the expression of TPH1 and 5-HT, in response to high dose of sodium butyrate [51–53]. Our study yielded similar results, as sodium butyrate and the GPR43 activator both suppressed TPH1 and DDC expressions while promoting MAOB, ALDH1B1 and SLC6A4 expressions when comparing with the inhibitor of GPR43 (Fig. 4D, E). This suggests that GPR43 activation through sodium butyrate simultaneously reduces 5-HT synthesis and increases its degredation, leading to a decrease in 5-HT concentration (Fig. 4A).

Previous studies have reported the potential beneficial effects of 5-HT in UC patients, who commonly experience symptoms of depression. 5-HT in the central nervous system has been shown to have alleviating effects on depression [54–56]. However, our study suggests a contrasting role for 5-HT in the periphery. Due to the presence of the blood-brain barrier, 5-HT in periphery can only activate the HTRs outside the central nervous system to exert downstream regulatory effects. Notably, HTR1A and HTR3 have the ability to activate T lymphocytes, thereby influencing the inflammatory response [17, 18, 57]. HTR1A is predominantly located on the membrane of T lymphocytes in the intestinal associated lymphoid tissue, where it facilitates cell proliferation and activation, regulating the synthesis and secretion of cytokines including IFN-γ, IL-1 and IL-2 [17]. Furthermore, HTR3 exhibits a wide distribution in both the central and intestinal nervous systems, regulating nerve conduction through membrane depolarization-induced Ca^2+^ influx [58]. Recent studies had revealed that the T lymphocyte activation by HTR3 depended on PKC signaling pathway and up-regulated the secretion of IL-1β, IL-6 and IL-18 in LPS-induced monocytes [17, 59, 60]. Our study observed consistent changes in the concentrations of IL-1β, IL-2, IL-6, TNF-α and IFN-γ with alterations in 5-HT concentrations (Figs. 1F, 5F) and PKCθ and NF-κB expressions (Fig. 5D, E). These results suggest that 5-HT-mediated activation of downstream T lymphocytes contributes to inflammation in the colonic tissues of DSS-induced mice. Furthermore, the use of inhibitors targeting HTR1A and HTR3 would suppress T lymphocyte activation (Fig. 5D-F), underscoring the significance of these two HTRs. However, SYD can reduce the local colonic concentration of 5-HT and effectively alleviate colonic inflammation by suppressing T lymphocyte activation.

In conclusion, SYD modulates the intestinal flora to inhibit the gene expression for 5-HT synthesis in its genome directly and increases the intestinal flora-derived metabolites - butyric acid - to inhibit the synthesis of 5-HT, as well as prompt the transport and degredation of 5-HT, in the organism (Fig. 6). Consequently, the decrease in local colonic 5-HT levels caused by SYD alleviates colonic inflammation by regulating T lymphocyte activation and cytokine synthesis through HTRs (Fig. 6).

## Conclusion

SYD ameliorated colonic inflammation in DSS-induced mice by controlling T lymphocyte activation through the modulation of 5-HT metabolism in the local colon, mediated by the intestinal flora and its metabolites.

### Supplementary Information

Supplementary Material 1.

Supplementary Material 2.

## Data Availability

The original datasets used during the current study are available from the corresponding author on reasonable request. The datasets generated and analyzed during the current study are available from the supplementary tables.

## Abbreviations

5-HT: 5-Hydroxytryptamine
ALDH1B1: Aldehyde dehydrogenase 1 family member B1
ANOVA: Analysis of variance
ATCC: American type culture collection
BSA: Bovine serum albumin
DAB: Diaminobenzidine
DAI: Disease activity index
DAPI: 4',6-Diamidino-2-phenylindole
DDC: Dopa decarboxylase
DEGs: Differentially expressed genes
DSS: Dextran sulfate sodium
DW: Distilled water
ECCs: Enterochromaffin cells FBS Fetal bovine serum
ELISA: Enzyme linked immunosorbent assay
FBS: Fetal bovine serum
FC: Fold change
FPKM: Fragments per kilobase of transcript per million fragments mapped
GAPDH: Glyceraldehyde-3-phosphate dehydrogenase
GPR: G protein-coupled receptor
GPR43: G protein-coupled receptor 43
HTR1A: 5-HT receptor 1A
HTR2A: 5-HT receptor 2A
HTR1A: 5-HT receptor 1A
HTR3: 5-HT receptor 3
HE: Hematoxylin-eosin
IF: Immunofluorescence
IFN-γ: Interferon-gamma
IHC: Immunohistochemistry
IL-1β: Interleukin-1 beta
IL-2: Interleukin-2
IL-6: Interleukin-6
JAK2: Janus kinase 2
KEGG: Kyoto Encyclopedia of Genes and Genomes
LC: Liquid chromatography
LDA: Linear discriminant analysis
LEfSe: Linear discriminant analysis effect size
MAOA: Monoamine oxidase A
MAOB: Monoamine oxidase B
MS: Mass spectrum
NC: Normal control
NF-κB: Nuclear factor kappa-B
OTUs: Operational taxonomic units
PANTHER: Protein Analysis Through Evolutionary Relationships
PBST: Phosphate buffered saline with tween
PVDF: Polyvinylidene fluoride
PCoA: Principal coordinates analysis PICRUSt Phylogenetic Investigation of Communities by Reconstruction of Unobserved States
PICRUSt: Phylogenetic Investigation of Communities by Reconstruction of Unobserved States
PKCθ: Protein kinase C theta
QIIME: Quantitative Insights Into Microbial Ecology
RIPA: Radio immunoprecipitation assay
RT-qPCR: Real time-quantitative polymerase chain reaction
SCFAs: Short-chain fatty acids
SDS-PAGE: Sodium dodecyl sulfate-polyacrylamide gel electrophoresis
SLC6A4: Sodium-dependent serotonin transporter
SPSS: Statistical Package for Social Sciences
STAT3: Signal transducer and activator of transcription 3
SYD: Shaoyao Decoction
SYD-H: High dose of Shaoyao Decoction
SYD-L: Low dose of Shaoyao Decoction
SYD-M: Medium dose of Shaoyao Decoction
TCM: Traditional Chinese Medicine
TCMSP: Traditional Chinese Medicine Systems Pharmacology Database and Analysis Platform
TNF-α: Tumor necrosis factor-α
TPH1: Tryptophan hydroxylase 1
TCM: Traditional Chinese Medicine
TBST: Tris buffered saline with tween
UC: Ulcerative colitis
UMI: Unique molecular identifier
UPLC-MS/MS: Ultra-performance liquid chromatography tandem mass spectrometry
WB: Western blot
