# Supplementary material for: Shaoyao Decoction reduced T lymphocyte activation by regulating of intestinal flora and 5-hydroxytryptamine metabolism in ulcerative colitis
Source: Chin Med. 2024 Jun 15;19:87. doi: 10.1186/s13020-024-00958-2 (PMC11180410; doi:10.1186/s13020-024-00958-2)

**Supplementary Fig. 1:** The visceral organ indexes and WB analysis in the mice experiment. (A) Thymus and spleen indexes in SYD treating DSS-induced mice. (B) WB analysis of PKCθ and NF-κB in the colonic tissue. Protein expression levels were normalized to GAPDH levels. 3 mice per group. Compared with NC, ^*^ *P* ≤ 0.05, ^**^ *P* ≤ 0.01; compared with M, ^#^ *P* ≤ 0.05, ^##^ *P* ≤ 0.01; compared with SYD-L, ^&^ *P* ≤ 0.05, ^&&^ *P* ≤ 0.01; compared with SYD-M, ^▲^ *P* ≤ 0.05, ^▲▲^ *P* ≤ 0.01; compared with SYD-H, ^●^ *P* ≤ 0.05, ^●●^ *P* ≤ 0.01.


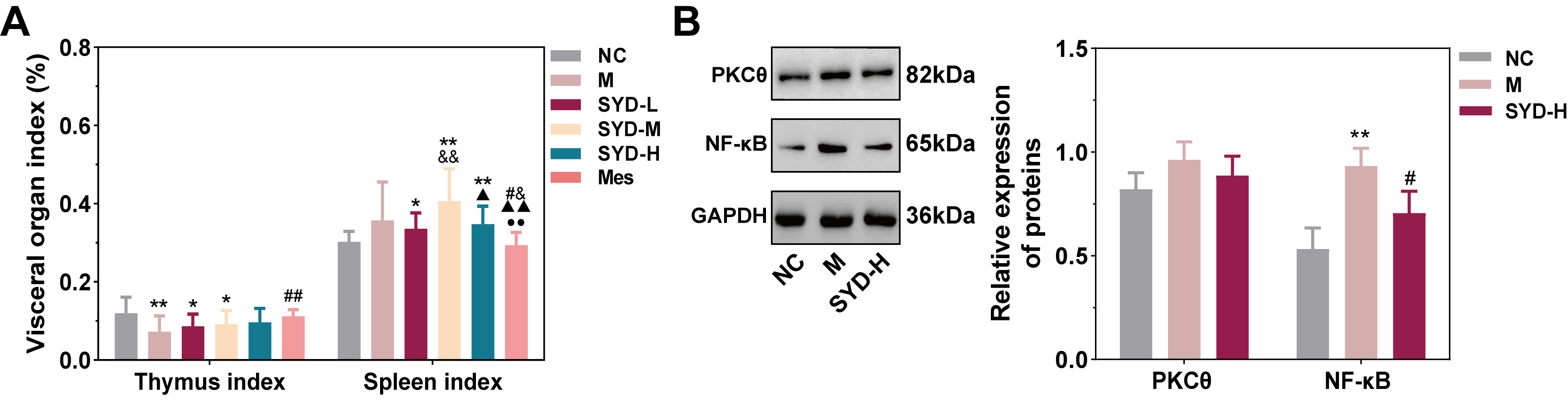


**Supplementary Fig. 2:** Ingredients of SYD in UPLC-MS/MS, and PANTHER pathways enriched by the targeted genes related to the ingredients of SYD in UPLC-MS/MS. (A) SYD in positive ion mode of UPLC-MS/MS. (B) Berberine or epiberberine in SYD in positive ion mode of UPLC-MS/MS - 10.00 min. (C) Berberine or epiberberine in SYD in positive ion mode of UPLC-MS/MS - 10.14 min. (D) Baicalin or isomer in SYD in positive ion mode of UPLC-MS/MS - 8.91 min. (E) SYD in negative ion mode of UPLC-MS/MS. (F) Baicalin in SYD in negative ion mode of UPLC-MS/MS - 26.11 min. (G) Wogonin in SYD in negative ion mode of UPLC-MS/MS - 29.78 min. (H) Paeoniflorin in SYD in negative ion mode of UPLC-MS/MS - 15.87 min. (I) Content of paeoniflorin, baicalin and berberine quantified by UPLC-MS/MS. (J) PANTHER pathway enrichment analysis based on the targeted genes related to ingredients of SYD in UPLC-MS/MS. The horizontal axis represents the *P*. The size of the circle represents the gene number in the enrichment analysis.


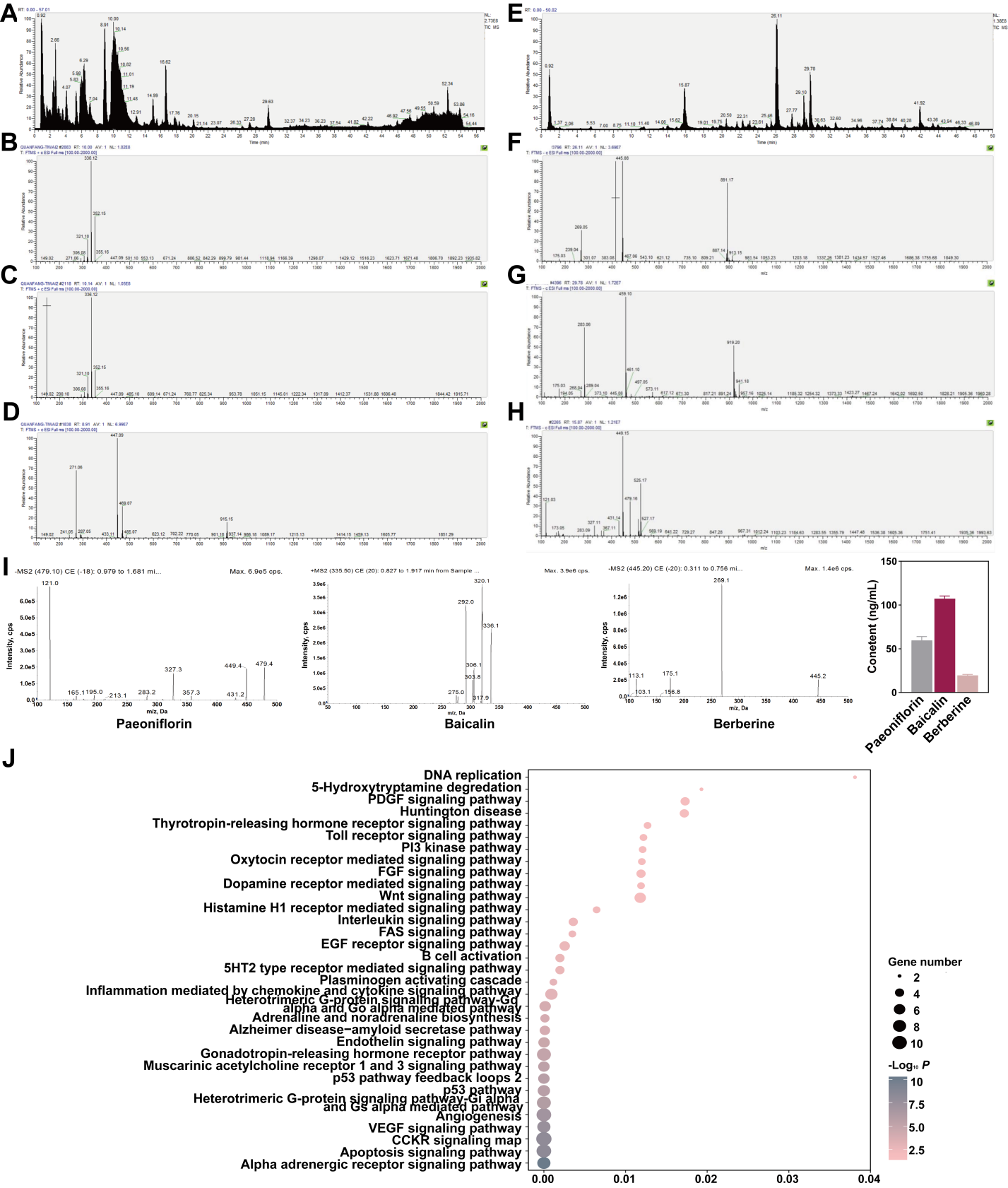


**Supplementary Fig. 3:** DEGs in the colonic tissue, and the core enriched KEGG/PANTHER pathways in SYD treating DSS-induced mice. (A) PCoA based on all gene expression profile (Adonis: R^2^ = 0.499, *P* = 0.000). (B) Distribution of DEGs shown in volcano plot. Threshold was set to be “|log_2_FC| ≥ 1.00 and *P* ≤ 0.05”. (C) The distribution of DEGs. (D) Methods that core enriched KEGG/PANTHER pathways based on multiple DEGs sets. (E) Core KEGG pathways that played important roles in SYD-H treatment. (F) Schematic diagram of 5-HT synthesis/degredation and the key enzymes based on PANTHER pathways.


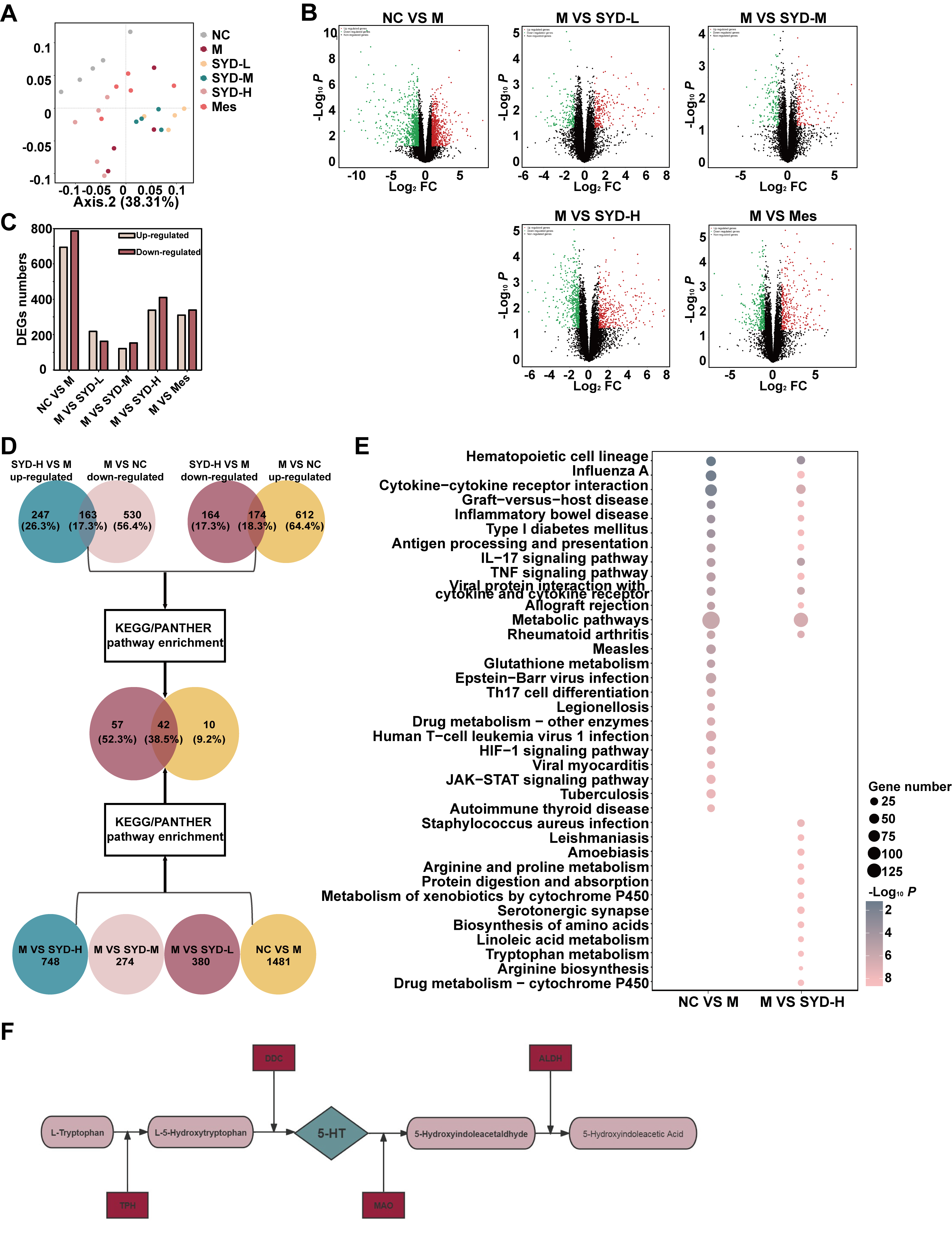


**Supplementary Fig. 4:** SYD regulated the intestinal flora. Kruskal-Wallis tests of (A) alpha indexes and (B) beta distances. (C) PCoA based on Bray-Curtis distance (Adonis: R^2^ = 0.411, *P* = 0.000). (D) The differential genera by taking intersection of the Kruskal-Wallis test and the LEfSe analysis (LDA ≥ 2 and *P* ≤ 0.05). *P* was obtained by the Kruskal-Wallis test. Compared with NC, ^*^ *P* ≤ 0.05, ^**^ *P* ≤ 0.01.


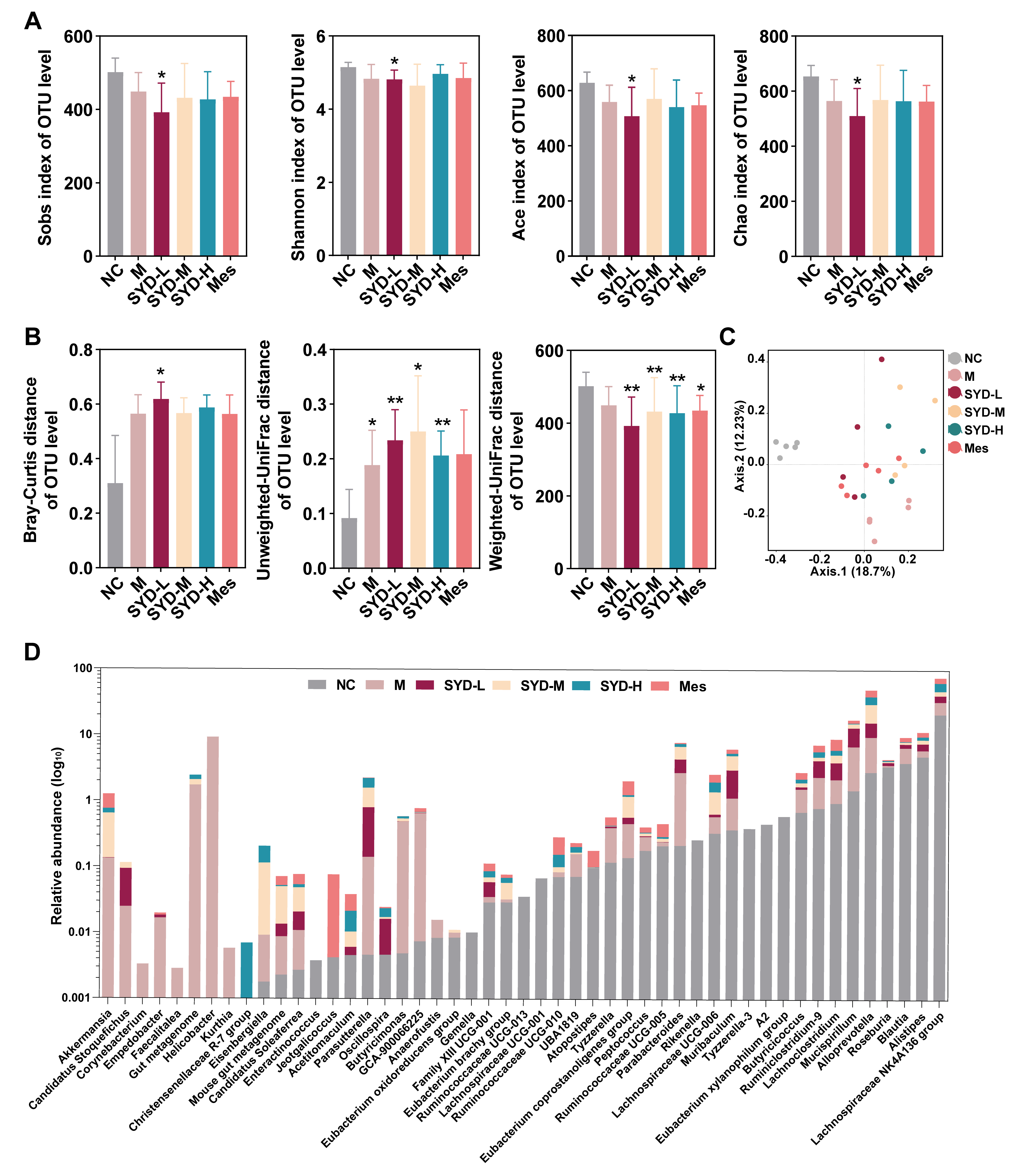


**Supplementary Fig. 5:** Heatmap based on the Spearman’s correlation between 5-HT concentration in the faeces and SCFAs production in the faeces/serum. The SCFAs in the green box were in the faeces, while SCFAs in the red box were in the serum. Red represents the positive correlation and green represents the negative correlation. ^*^ *P* ≤ 0.05, ^**^ *P* ≤ 0.01.


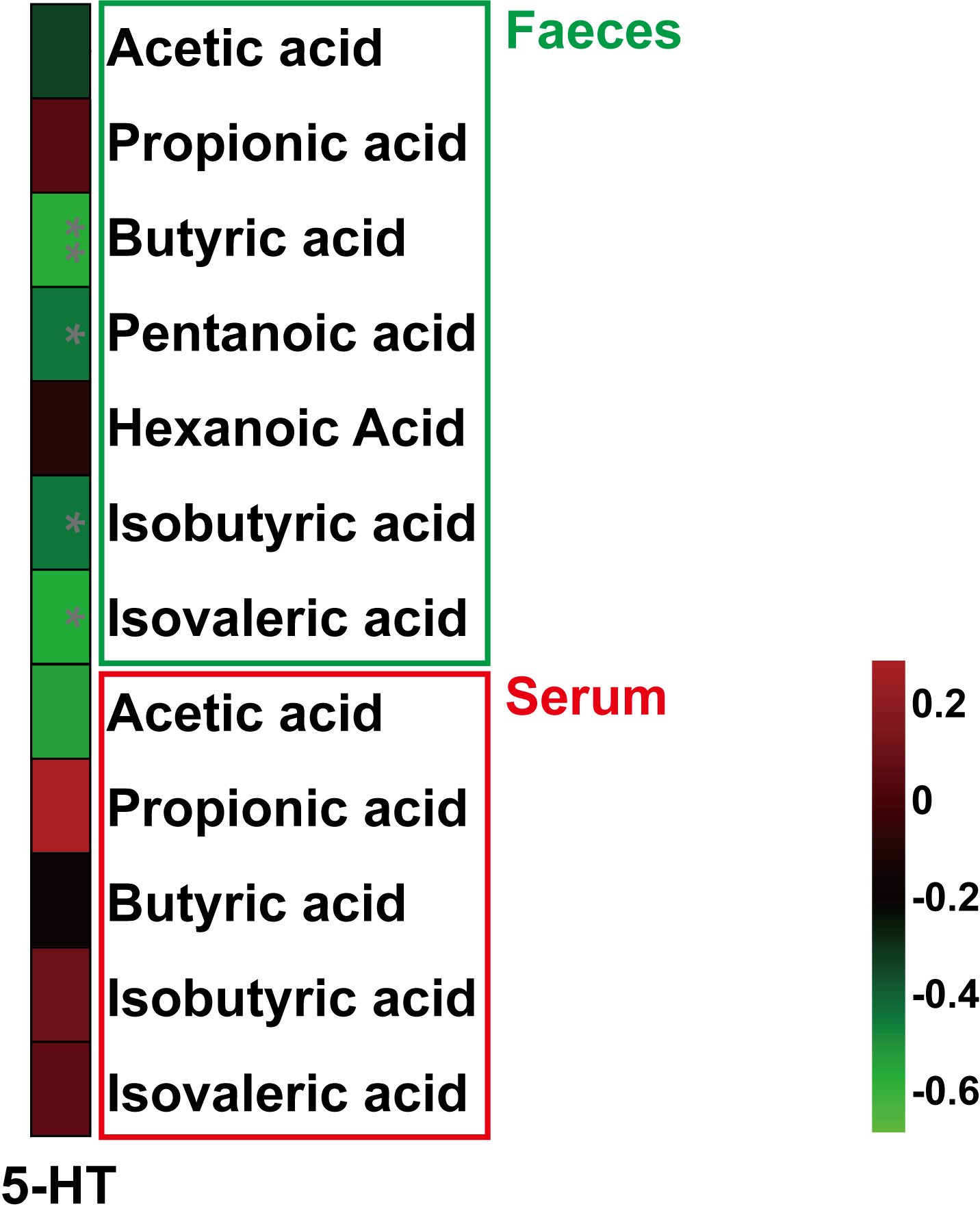


**Supplementary Fig. 6:** Determination of cell viability. (A) The effect of different concentrations of L-Trp, sodium butyrate (SB), GLPG0974 and TUG-1375 on QGP-1 cell viability. (B) The effect of different concentrations of 5-HT, Way100635 and Tropisetron on H9 cell viability. ^*^ *P* ≤ 0.05.


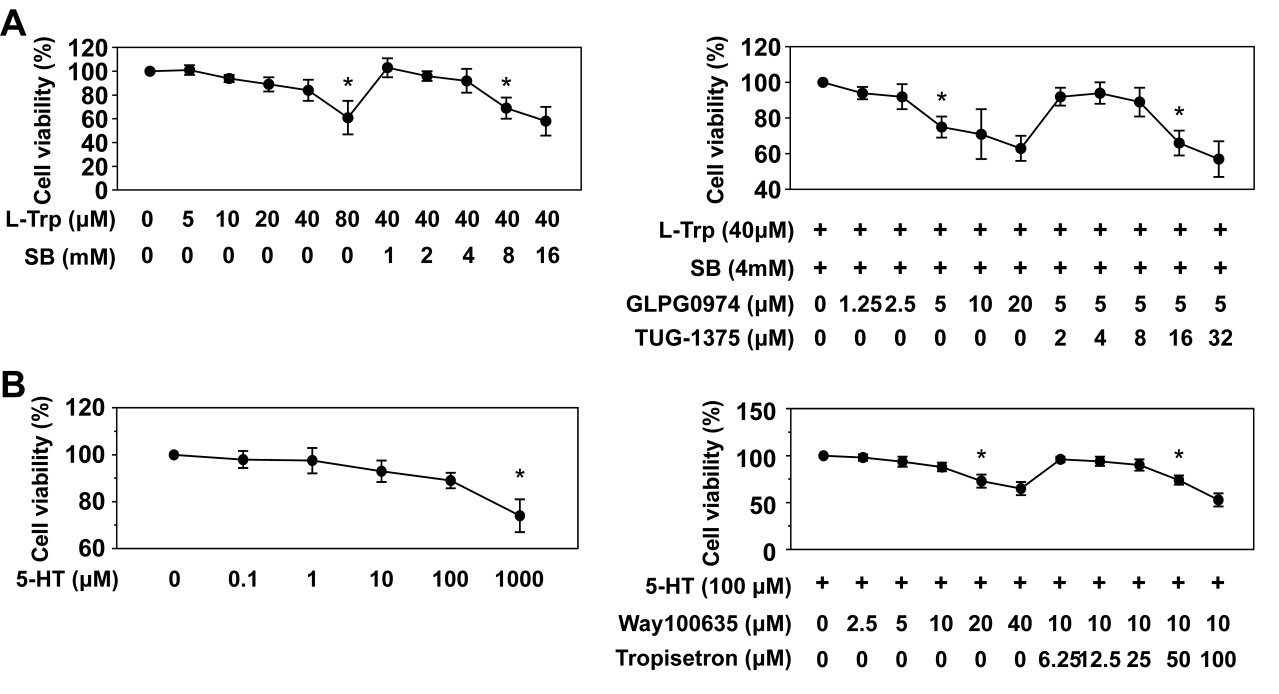


**Supplementary Fig. 7:** Spearman’s correlation between genes related to 5-HT metabolism in faeces and the key differential genera. Red represents the positive correlation and green represents the negative correlation. ^*^ *P* ≤ 0.05, ^**^ *P* ≤ 0.01, ^***^ *P* ≤ 0.001.


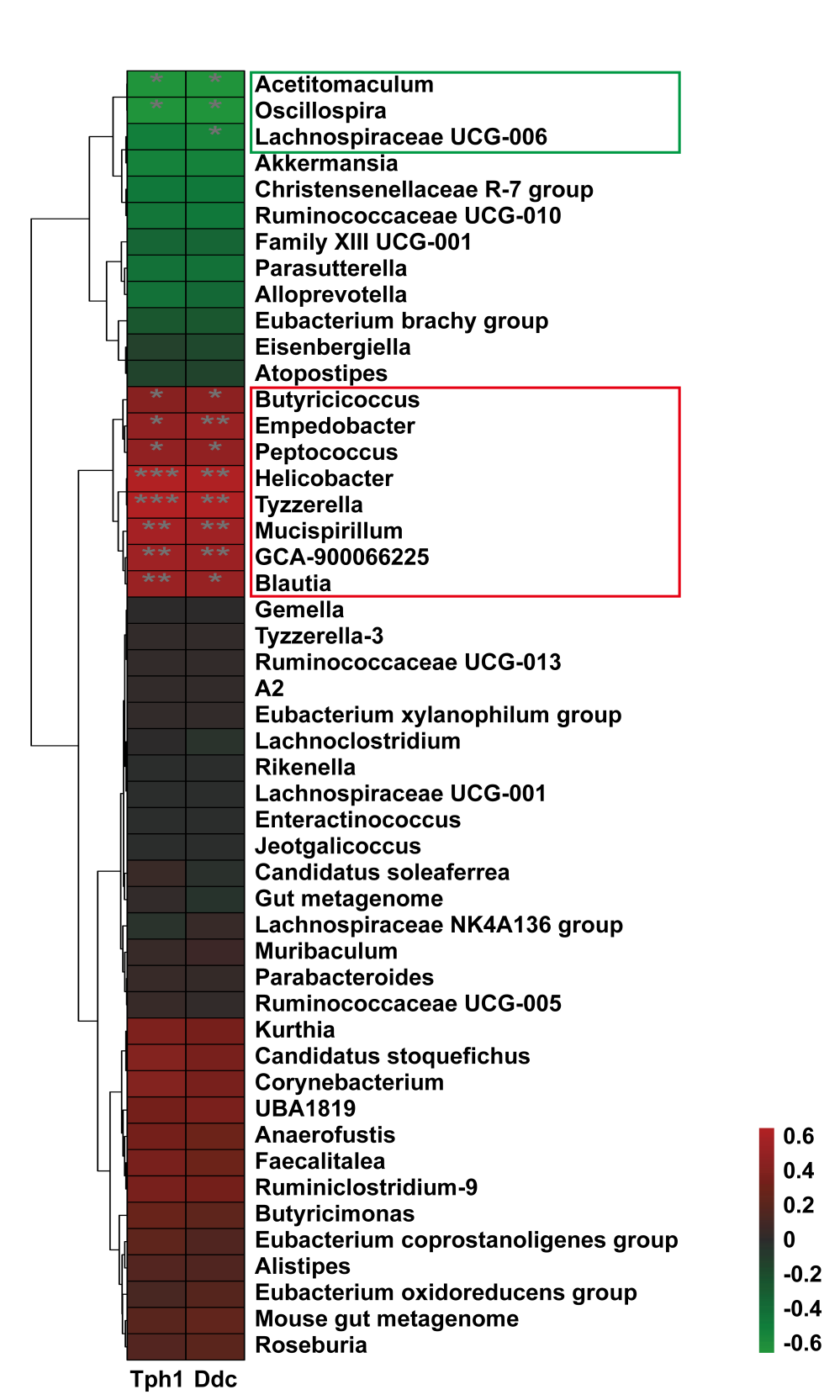

Supplement: Supplementary file 2 — Supplementary Material 2. [file 13020_2024_958_MOESM2_ESM.docx]
